# The use of urinary lateral-flow lipoarabinomannan assays for TB diagnosis in children

**DOI:** 10.5588/ijtldopen.25.0667

**Published:** 2026-05-11

**Authors:** I. Sabi, L. Olbrich, W. Olomi, A. Mfinanga, H.J. Zar, M.P. Nicol, Z. Franckling-Smith, C. Khosa, D. Banze, M. Nliwasa, E.L. Corbett, R. Semphere, V.P. Verghese, J.S. Michael, L. Larsson, S.M. Graham, R. Song, P. Nabeta, A. Trollip, M. Hoelscher, C. Geldmacher, N.E. Ntinginya, N. Heinrich, on behalf of the RaPaed-AIDA-TB Consortium

**Affiliations:** 1 Mbeya Medical Research Centre, National Institute for Medical Research, Mbeya, Tanzania;; 2 Department of Paediatrics and Child Health, School of Medicine and Dentistry, University of Dodoma, Dodoma, Tanzania;; 3 Institute of Infectious Diseases and Tropical Medicine, LMU University Hospital, Ludwig Maximilian University of Munich, Munich, Germany;; 4 Fraunhofer Institute for Translational Medicine and Pharmacology, Immunology, Infection and Pandemic Research, Munich, Germany;; 5 German Centre for Infection Research (DZIF), Partner Site Munich, Munich, Germany;; 6 Department of Paediatrics and Child Health, SA-MRC Unit on Child and Adolescent Health, University of Cape Town, Cape Town, South Africa;; 7 Marshall Centre, School of Biomedical Sciences, University of Western Australia, Perth, WA, Australia;; 8 Instituto Nacional de Saúde (INS), Marracuene, Mozambique;; 9 Department of Physiological Science, Clinical Pharmacology, Faculty of Medicine, Eduardo Mondlane University, Maputo, Mozambique;; 10 Departments of Clinical Science and International Public Health, Liverpool School of Tropical Medicine, Liverpool, UK;; 11 TB/HIV Research Group, College of Medicine, Blantyre, Malawi;; 12 TB Centre, London School of Hygiene and Tropical Medicine, London, UK;; 13 Pediatric Infectious Diseases, Department of Pediatrics, Christian Medical College (CMC), Vellore, India;; 14 Department of Clinical Microbiology, Christian Medical College (CMC), Vellore, India;; 15 University of Melbourne Department of Paediatrics and Murdoch Children’s Research Institute, Royal Children’s Hospital, Melbourne, VIC, Australia;; 16 Oxford Vaccine Group, Department of Paediatrics and National Institute for Health and Care Research Oxford Biomedical Research Centre, University of Oxford, Oxford, UK;; 17 FIND (Foundation for Innovative New Diagnostics), Geneva, Switzerland.

**Keywords:** tuberculosis, paediatric TB, LAM, FujiLAM, South Africa, Tanzania, Mozambique, Malawi, India

## Abstract

**BACKGROUND:**

Detection of lipoarabinomannan (LAM) in urine is a promising non-sputum-based test for TB. We aimed to evaluate Fujifilm SILVAMP TB-LAM (FujiLAM) for the diagnosis of TB in children, using AlereLAM as a comparator.

**METHODS:**

Between January 2019 to July 2021, children and young adolescents with presumptive TB disease were enrolled in five countries as part of the RaPaed-TB study. Standardised microbiological, radiologic, and clinical data were used to define TB category following published consensus criteria. FujiLAM and AlereLAM were performed on a single urine sample at enrolment.

**RESULTS:**

Of 975 children enrolled, 818 (83.9%) had FujiLAM results available. Median age was 5.5 years (interquartile range 2.4–9.4), 83 (10.1%) had severe acute malnutrition, and 138 (16.9%) were living with HIV. Using a microbiological reference standard, overall sensitivity of FujiLAM was 31.0% (95% confidence interval [CI]: 24.7–37.9) and that of AlereLAM 13.3% (95% CI: 9.0–18.8), whereas the overall specificity was 89.8% (95% CI: 86.3–92.7) and 92.7% (95% CI: 89.6–95.1), respectively. Marked differences in FujiLAM accuracy were noted between production lots.

**CONCLUSION:**

FujiLAM remains a promising non-invasive, urine-based biomarker for childhood TB diagnosis. However, the observed lot-to-lot variability underscores the need for improved assays with rigorous multi-lot evaluations to ensure reliability in future studies.

The diagnosis of TB disease in children is challenging, despite concerted efforts to develop new diagnostic tools. Microbiological confirmation is challenging and existing diagnostics perform sub-optimally in children, resulting in many undiagnosed, untreated, and unreported cases.^1–3^ The WHO has prioritised the development of novel point-of-care tests that can diagnose TB regardless of age, HIV, or nutritional status.^4,5^ Urine lipoarabinomannan (LAM) detection offers a promising non-sputum target for TB diagnosis. Currently, AlereLAM (Abbott, Palatine, IL, USA) is the only commercially available lateral-flow immunoassay, but its sensitivity is sub-optimal, and limited to people living with HIV (PLHIV) with TB symptoms or advanced HIV.^6^ The Fujifilm SILVAMP TB-LAM assay (FujiLAM; Fujifilm, Tokyo, Japan) is a newer, more sensitive test under development. Early studies in hospitalised adult PLHIV with TB showed higher FujiLAM sensitivity (70.0%) versus AlereLAM (42.3%).^7^ Modelling in South Africa predicted that a future LAM test with a sensitivity similar to FujiLAM has the potential to avert 29.6% of TB deaths and 17.7% of incident cases in people with TB symptoms regardless of HIV status.^8^ However, evidence in children remains insufficient^9–11^; a systematic review found FujiLAM more sensitive than AlereLAM in children, but more prospective data are needed.^12^

In this study, we prospectively tested FujiLAM in a paediatric diagnostic TB cohort in five low- and middle-income countries (LMICs) to evaluate the diagnostic accuracy of FujiLAM, using AlereLAM as a comparator.

## METHODOLOGY

This diagnostic accuracy study was part of the RaPaed-TB study (ClinicalTrials.gov, *NCT03734172*) in five high-TB-incidence countries (South Africa, Tanzania, Mozambique, Malawi, and India).^13,14^ Children and young adolescents (<15 years of age) with presumptive TB were enrolled. Children who were critically ill, with a body weight <2 kg, or on TB medication for more than 72 h (treatment or prophylaxis) were excluded. After enrolment, all children attended follow-up visits at month 1 and month 3; those who started on TB treatment or were still clinically unwell at month 3 had an additional visit at month 6.

### Study procedures

Participants’ demographic and clinical data were recorded, including medical history, physical examination, and HIV testing according to local guidelines. Tuberculin skin test, chest radiography, and two respiratory samples for TB reference standard testing were collected by sputum induction (African centres) or gastric aspirate (Indian centre). Extra-pulmonary samples were obtained when indicated. Samples underwent Xpert MTB/RIF Ultra® assay (Ultra, Cepheid Inc., USA), liquid culture (MGIT, BACTEC MGIT 960, Becton Dickinson), and solid culture (Löwenstein–Jensen) testing. Positive acid-fast bacilli were confirmed using the MPT64 Ag test and GenoType MTBDRplus V2® line-probe assay (Hain Lifescience, Germany).^15^ Urine samples were collected into sterile containers or using urine bags for nappy-wearing children following cleaning the area, and stored at −20°C when immediate testing was not possible. Both FujiLAM and AlereLAM were performed during the first visit according to manufacturer’s instructions. FujiLAM used approximately 200 µL of urine, involving five steps, taking up to 60 min as previously described.^7,11^ Any visible test line was considered positive. AlereLAM used 60 µL of urine, incubated for 25 min, and was visually interpreted. Any positive result was considered and grading was documented. To minimise bias for AlereLAM grading, AlereLAM was always performed and read before FujiLAM, and laboratory staff were blinded to participants’ clinical and microbiological results.

### Reference standards

Diagnostic classifications followed adapted NIH-consensus clinical case definitions for paediatric TB diagnostic studies (Supplementary Data Table S1).^15^ Reference standards were derived based on clinical case definitions: a Microbiological Reference Standard (MRS) comparing ‘confirmed TB’ (culture and/or Xpert Ultra, MRS positive) to ‘unlikely TB’ (MRS negative) and a Composite Reference Standard (CRS) comparing ‘confirmed’ and ‘unconfirmed TB’ (CRS positive) with ‘unlikely TB’ (CRS negative).

### Statistical analysis

To describe the study population, we present percentages for categorical variables and medians with interquartile ranges (IQRs) for continuous variables. In the diagnostic accuracy analysis, we calculated sensitivity, specificity, and predictive values for the investigational assays against the defined reference standard (i.e., MRS and CRS). We also performed subgroup analyses stratified by population and determinants (e.g., age, study sites, HIV status, and urine collection methods). The McNemar’s test for paired samples was used to compare the sensitivity and specificity of FujiLAM and AlereLAM, and the χ^2^ test to compare independent proportions. To ascertain factors associated with FujiLAM and AlereLAM positivity, a multilevel mixed-effects logistic regression model with random intercepts for country was conducted. First, univariable regression models were fitted for each covariate, and variables with *P* < 0.20 or those producing >10% change in the effect estimate for the main exposure were included in the multivariable model. Crude odds ratio and multivariable adjusted odds ratio, along with 95% confidence intervals (CIs) and *P* values, were reported. To identify if there is an underlying temporal trend in the dataset without requiring the specification of any given model, locally weighted scatter plot smoothing (LOWESS) was used to graphically represent the association between sensitivity and specificity and time as a proxy of lot number. A *P* value <0.05 was considered statistically significant. Statistical analyses were performed using Stata (version 17.0, StataCorp, College Station, TX) and R statistical software.

### Ethical statement

This study was performed following the study protocol and the Declaration of Helsinki.^16^ The protocol and the informed consent documents used in this study were approved by the coordinators and all sites’ institutional review boards. Written consent was provided by parents or guardians, with witnessed oral consent for illiterate participants. Older children provided assent according to local guidelines.

## RESULTS

**Figure 1.**
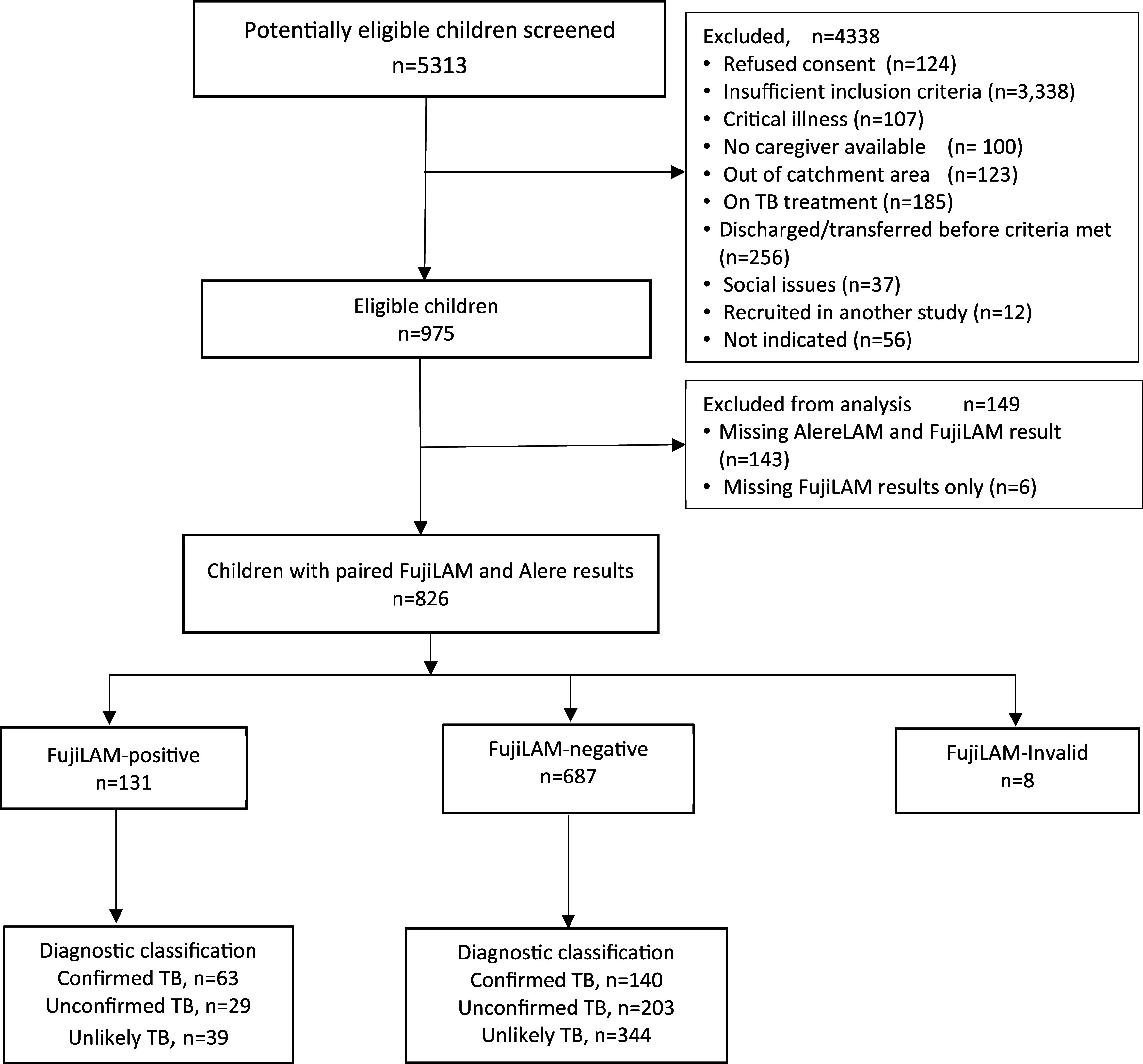
STARD diagram with FujiLAM test results by TB case definitions.

From January 2019 to July 2021, a total of 5,313 potentially eligible children were screened, and 975 were enrolled. Among those, 149 were excluded because no FujiLAM or AlereLAM tests were performed. In 826 with paired FujiLAM and AlereLAM test results, another 8 participants were excluded because of invalid FujiLAM results. Thus, 818 (83.9%) children were included in this analysis, of which 24.8% (203/818) were categorised as confirmed, 28.4% (232/818) as unconfirmed, and 46.8% (383/818) as unlikely TB (Figure 1). The median age was 5.5 years (IQR 2.4–9.4) and 438 (53.5%) were male. Overall, 138 (16.9%) were children living with HIV; of those, 43.9% (58/132) were ART-naïve or on ART <1 month at enrolment, and 10.1% (83/818) of the children had severe acute malnutrition (SAM) (Table, Supplementary Data Table S2).

**Table.** Baseline and clinical characteristics of study participants by diagnostic classification.

Characteristics	Confirmed TB	Unconfirmed TB	Unlikely TB	Total
	N = 203	N = 232	N = 383	N = 818
Men, n (%)	113 (55.7%)	132 (56.9%)	193 (50.4%)	438 (53.5%)
Age in years, median (IQR)	7.2 (2.4–11.9)	5.4 (2.4–9.0)	5.3 (2.5–8.2)	5.5 (2.4–9.4)
Age in years, n (%)				
<5	74 (36.5%)	108 (46.6%)	184 (48.0%)	366 (44.7%)
5 to <10 years	53 (26.1%)	74 (31.9%)	138 (36.0%)	265 (32.4%)
10 to <15 years	76 (37.4%)	50 (21.6%)	61 (15.9%)	187 (22.9%)
HIV status, n (%)				
HIV uninfected	181 (89.2%)	166 (71.9%)	316 (82.5%)	663 (81.2%)
HIV infected	21 (10.3%)	63 (27.3%)	54 (14.1%)	138 (16.9%)
Unknown	1 (0.5%)	2 (0.9%)	13 (3.4%)	16 (2.0%)
Malnutrition, n (%)				
SAM	27 (13.3%)	25 (10.8%)	31 (8.1%)	83 (10.1%)
MAM	26 (12.8%)	27 (11.6%)	43 (11.2%)	96 (11.7%)
No malnutrition	150 (73.9%)	180 (77.6%)	309 (80.7%)	639 (78.1%)
Country, n (%)				
South Africa	59 (29.1%)	58 (25.0%)	46 (12.0%)	163 (19.9%)
Tanzania	37 (18.2%)	47 (20.3%)	112 (29.2%)	196 (24.0%)
Mozambique	19 (9.4%)	68 (29.3%)	91 (23.8%)	178 (21.8%)
Malawi	32 (15.8%)	55 (23.7%)	105 (27.4%)	192 (23.5%)
India	56 (27.6%)	4 (1.7%)	29 (7.6%)	89 (10.9%)

IQR = interquartile range; SAM = severe acute malnutrition – defined as weight-for-height below −3 Z scores; MAM = moderate acute malnutrition – defined as weight-for-height below −2 Z scores.

**Figure 2.**
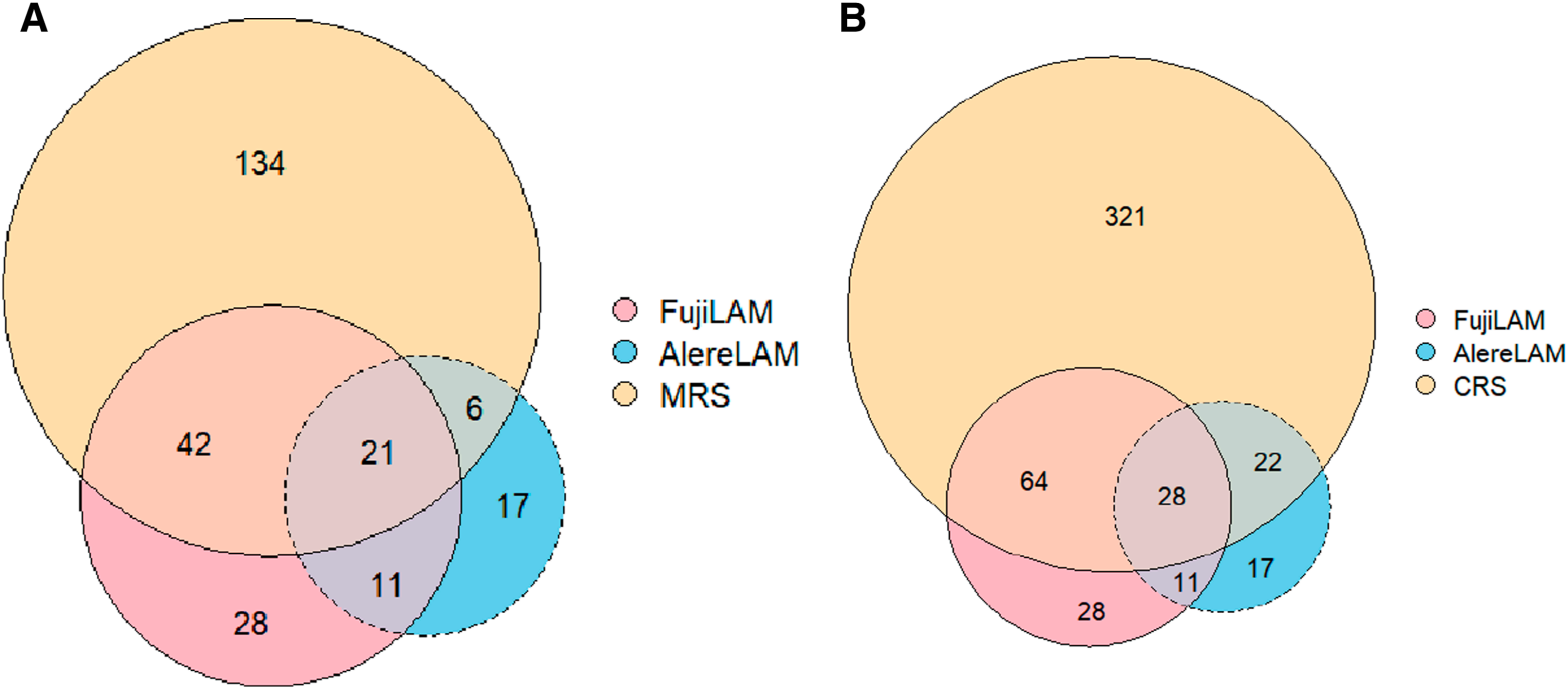
**A:** Three-way Venn diagram of FujiLAM, AlereLAM, and MRS results; **B:** Three-way Venn diagram of FujiLAM, AlereLAM, and CRS results. MRS = microbiological reference standard; CRS = composite reference standard.

Figure 2 visualises the head-to-head comparison of FujiLAM and AlereLAM. Using the MRS (confirmed TB vs. unlikely TB), n = 203 children were classified as MRS positive. Of those, 134/203 (66.0%) tested negative on both FujiLAM and AlereLAM, 42/203 (20.7%) were positive on FujiLAM only, and 6/203 (3.9%) on AlereLAM only, while 21/203 (10.3%) children were positive on both. Of those children classified as MRS negative, 28/383 (7.3%) were only positive in FujiLAM, 17/383 (4.4%) only using AlereLAM, and 11/383 (2.9%) were positive in both (Figure 2A). Using CRS, n = 435 children were identified as CRS positive. Of those, 321/435 (73.8%) tested negative on both FujiLAM and AlereLAM, 64/435 (14.7%) were positive on FujiLAM only, and 22/435 (5.1%) on AlereLAM only, while 28/435 (6.4%) children were positive on both FujiLAM and AlereLAM (Figure 2B).

**Figure 3.**
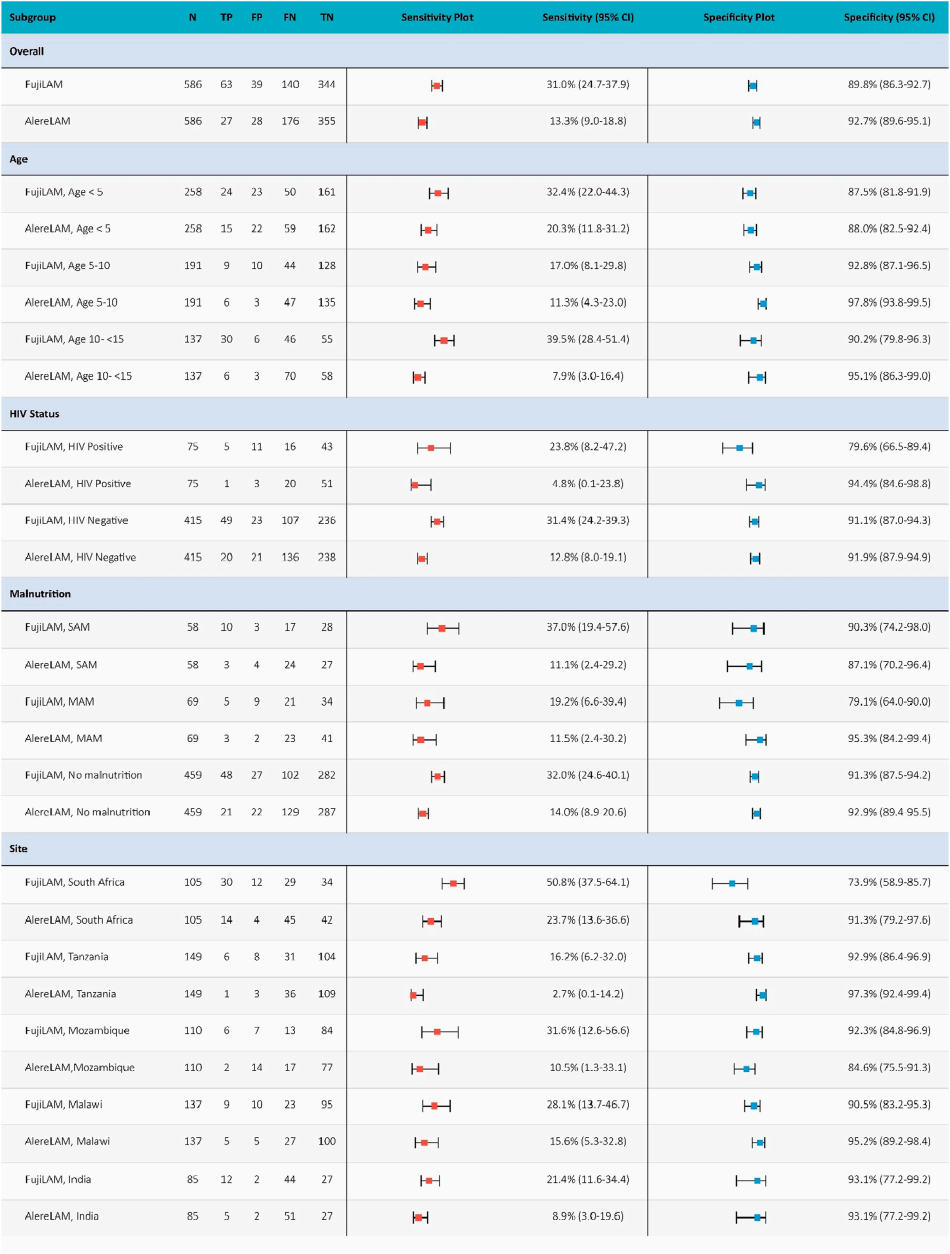
Sensitivity, specificity, and predictive values of Fujifilm SILVAMP TB-LAM in children with ‘microbiological confirmed TB’ and ‘Unlikely TB’ (MRS). TP = true positive; FP = false positive; TN = true negative; FN = false negative; SAM = severe acute malnutrition; MAM = moderate acute malnutrition.

Using the MRS, the overall sensitivity of FujiLAM was 31.0% (95% CI: 24.7–37.9) and that of AlereLAM was 13.3% (95% CI: 9.0–18.8), being significantly different (*P* < 0.0001). Overall specificity was 89.8% (95% CI: 86.3–92.7) for FujiLAM and 92.7% (95% CI: 89.6–95.1) for AlereLAM (Figure 3). By age group, the sensitivity of FujiLAM was highest and higher than that of AlereLAM in children aged 10 to <15 years (FujiLAM: 39.5%; 95% CI: 28.4–51.4 vs. AlereLAM: 7.9%; 95% CI: 3.0–16.4; *P* < 0.0001), and in children aged <5 years (FujiLAM: 32.4%; 95% CI: 22.0–44.3 vs. AlereLAM: 13.3%; 95% CI: 9.0–18.8, *P* = 0.04). Specificities were relatively similar across age groups and tests, ranging from 87.5% (95% CI: 81.8–91.9) to 92.8% (95% CI: 87.1–96.5) for FujiLAM and 88.0% (95% CI: 82.5–92.4) to 97.8% (95% CI: 93.8–99.5) for AlereLAM.

By HIV status, FujiLAM had a higher sensitivity, which was significantly greater than that of AlereLAM, among HIV-uninfected children (FujiLAM: 31.4%, 95% CI: 24.2–39.3; AlereLAM: 12.8%, 95% CI: 8.0–19.1; *P* < 0.0001). Analysis by CD4-count was limited due to the small sample size (Supplementary Data Table S4). By nutrition status, the sensitivities for FujiLAM were higher, but lower for AlereLAM in children with SAM (FujiLAM: 37.0%; 95% CI: 19.4–57.6 vs. AlereLAM: 11.1%; 95% CI: 2.4–29.2; *P* = 0.02). The sensitivity of FujiLAM was also higher than that of AlereLAM in children with no malnutrition (FujiLAM: 32.0%; 95% CI: 24.6–40.1 vs. AlereLAM: 14.0%; 95% CI: 8.9–20.6; *P* < 0.001), although it remained lower than in those with SAM. Specificities were similar across all subgroups and for both assays.

When accuracy was assessed by country, FujiLAM demonstrated the highest sensitivity among South African children, which was significantly greater than that of AlereLAM (FujiLAM: 50.8%; 95% CI: 37.5–64.1 vs. AlereLAM: 14.5%; 95% CI: 8.8–22.0; *P* < 0.001). In other countries, FujiLAM sensitivities were lower, and with no statistically significant difference from AlereLAM. Notably, FujiLAM specificity was lowest in South African children (73.9%; 95% CI: 58.9–85.7), while differences in AlereLAM specificity across countries were less pronounced (Supplementary Data Table S4).

Accuracy was further assessed by clinical and operational characteristics (Supplementary Data Table S4). FujiLAM’s sensitivity was higher with same-day urine processing (36.3%; 95% CI: 27.8–45.4) than after storage (22.8%; 95% CI: 14.1–33.6), but with wide and overlapping CIs. The specificities remained similar regardless of processing time. FujiLAM’s sensitivity was higher with urine bags (58.3%; 95% CI: 27.7–84.8) than with catch urine (26.1%; 95% CI: 18.2–35.3), but with wide and overlapping CIs. AlereLAM sensitivity and specificities stayed comparable regardless of processing time.

Applying CRS, the overall sensitivity of FujiLAM (21.1%; 95% CI: 17.4–25.3) was lower compared to MRS but remained higher than that of AlereLAM (11.5%; 95% CI: 8.7–14.9), with similar specificity (Supplementary Data Table S5).

Determinants of LAM true positivity and negativity were assessed using logistic regression comparing MRS-positive children with positive versus negative LAM (Supplementary Data Tables S6–S9). The true positivity and true negativity of FujiLAM were associated with the lot numbers (19001, 20001, 20003, and 20004). Specifically, lot 20001 had lower odds of true positivity (OR = 0.09, 95% CI: 0.01–0.84), while lot 20004 had higher odds of true negativity (OR = 4.27; 95% CI: 1.08–16.83), both compared to lot 19001 as the reference.

**Figure 4.**
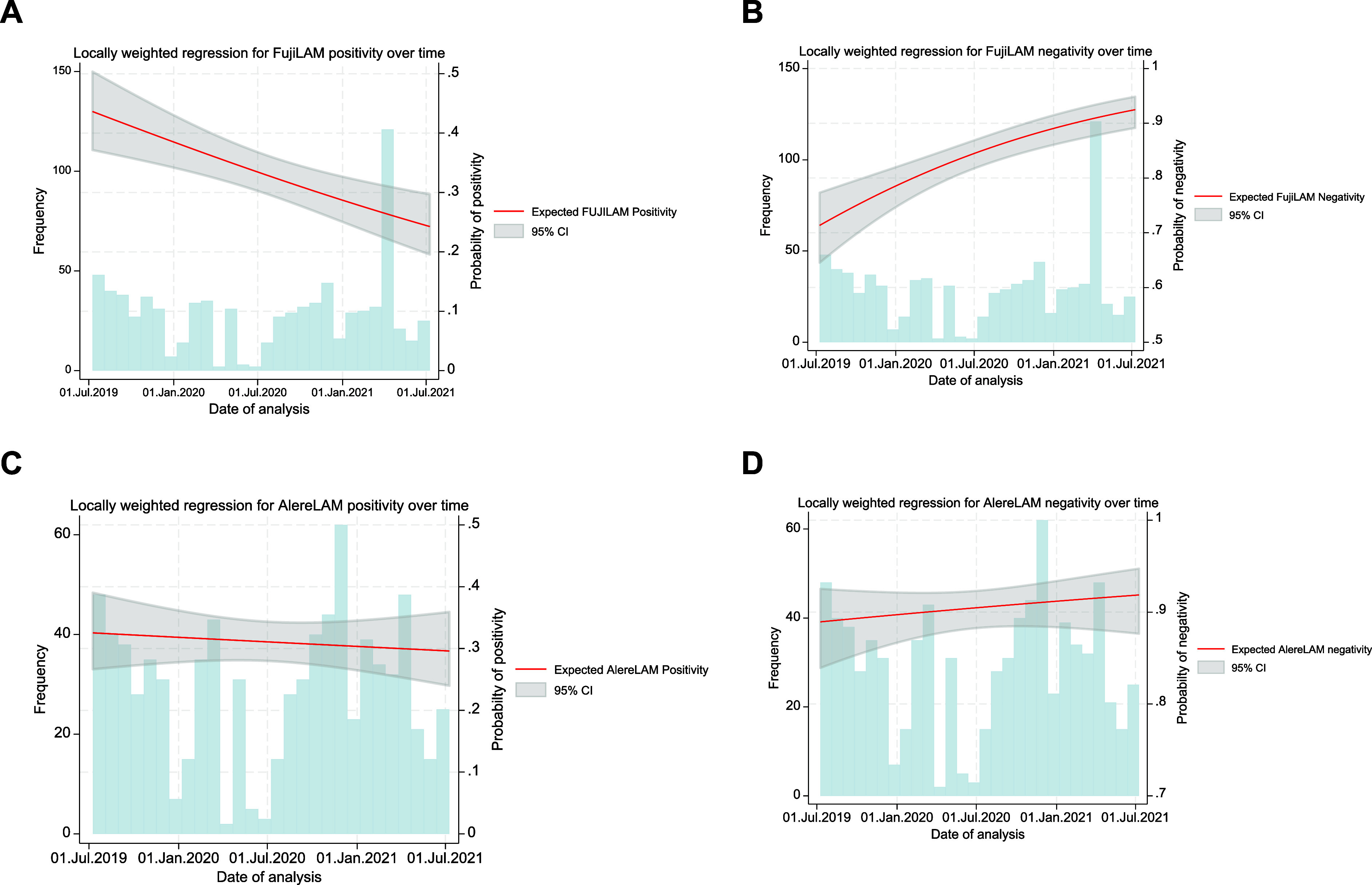
The LOWESS curve showing a trend relationship between the sensitivity and specificity over time for FujiLAM and AlereLAM, respectively. **A:** LOWESS curve indicating FujiLAM sensitivity decreasing over time; **B:** LOWESS curve indicating FujiLAM negativity increasing over time; **C:** LOWESS curve showing AlereLAM sensitivity remaining fairly constant over time; **D:** LOWESS curve showing AlereLAM specificity remaining fairly constant over time.

In a post-hoc analysis, we assessed the differences in test accuracy, examining FujiLAM and AlereLAM sensitivity and specificity trends over time using a locally weighted smoothing scatter plot (LOWESS). As FujiLAM lot numbers were not systematically recorded per test, time served as a proxy for lot variation. LOWESS curves stratified by site showed considerable differences, suggesting a temporal trend of decreasing sensitivity and increasing specificity over time (Figure 4; Supplementary Data Figure S1).

## DISCUSSION

In this evaluation of the performance of FujiLAM compared to AlereLAM in a large cohort of children with presumed TB across five LMICs, we found a higher sensitivity of FujiLAM compared to AlereLAM overall and in subgroups of interest. Accuracy estimates were similar across subgroups, with trends of higher sensitivity in HIV-uninfected children and those with SAM for FujiLAM. We did notice an overall low concordance between tests positivity between AlereLAM and FujiLAM. In addition, there were marked differences in estimated accuracy depending on geographic location and over time for FujiLAM, but not AlereLAM, suggesting a marked impact of production lot. In addition, both tests demonstrated sub-optimal sensitivity across all groups, falling short of WHO performance criteria for screening or diagnostic tests.

FujiLAM sensitivity in our study was lower than in previous studies in children, where sensitivity ranged from 42.0% to 63.0%.^12^ Differences in reference standards might explain these variations, as studies in childhood TB face methodology challenges due to imperfect reference standards. While all published FujiLAM accuracy studies in children applied NIH-consensus case definitions, the reference standard application and interpretation varied.^9–11^ Our results suggest similar or even higher sensitivities for both FujiLAM and AlereLAM in some subgroups, especially children <5 years and those with malnutrition. Nicol et al.^9^ reported FujiLAM and AlereLAM sensitivities of 61.9% and 66.7% in children with malnutrition. Malnutrition is a known risk factor for TB and for severe disease,^17^ supporting the idea that LAM tests may be useful for the evaluation of TB in malnourished children. When considering operational characteristics, FujiLAM showed higher sensitivity, but both tests had lower specificity in urine bag samples, potentially due to faecal contamination compared to catch urine.^18^

We noticed a low concordance of test positivity between AlereLAM and FujiLAM, suggesting that these assays, or at least one of them, might have little value. In a study evaluating FujiLAM compared to AlereLAM on the same urine sample in PLHIV, both assays were positive in 31.6% of those with microbiologically confirmed TB.^19^ An additional 28.1% were positive on FujiLAM only and 8.8% on AlereLAM only, overall suggesting a greater concordance between assays compared to our results.

Our findings suggest a marked impact of production lot on FujiLAM accuracy. Although lot numbers were not recorded per test, retrospective linkage using testing dates and lab notes indicated significant temporal variation in FujiLAM sensitivity and specificity, unlike AlereLAM. This aligns with previous reports of lot-to-lot variability from a large multicentre study (n > 1,600)^20^ and in a recent prospective evaluation of FujiLAM in PLHIV.^19^ Manufacturing optimisation of the FujiLAM assay to address lot-to-lot variability has reportedly been completed, but field evaluations remain limited. Adzemovic et al.^21^ assessed the modified Fujifilm SILVAMP TB-LAM assay (FujiLAM II) on frozen urine samples from adults with advanced HIV, yet only one production lot was used, and assay modifications were undisclosed. These limitations underscore the unresolved lot-to-lot variability issues and hinder robust interpretation of FujiLAM results.

Previous FujiLAM diagnostic accuracy studies in children used biobanked urine samples.^9–11^ Connely et al.^22^ demonstrated that freezing of unprocessed urine reduces LAM concentration, potentially lowering sensitivity. Broger et al.^23^ found high LAM stability after freezing but noted slightly higher sensitivity in fresh samples. In our study, half of the children had fresh urine tested; neither stratification nor logistic regression yielded strong evidence for significant associations, supporting evidence that freezing and storage likely have a limited impact on FujiLAM performance.^23^

This study has some limitations. Participants were largely recruited at tertiary referral hospitals and utilised rigorous definitions for presumptive TB cases that may limit the generalisability of our findings.^2^ Nevertheless, a strength was rigorous diagnostic processes, including microbiological testing to ensure a sufficient number of confirmed TB cases. Despite a large sample size, the number of confirmed TB cases in most subgroups was small, limiting the precision of results.

## CONCLUSION

Our findings underscore the potential use of LAM assays for TB diagnosis in children, and LAM remains a promising biomarker that offers the vital advantage of non-invasiveness and quick turnaround time. Urine, as a sample type, is easy to collect and well-suited for children, but improved tests for TB detection are needed. Our data suggest variations in performance estimates of FujiLAM across different production lots. This highlights the need for future studies to rigorously evaluate multiple lots of novel assays to ensure reliability and generalisability.

## Supplementary Material

## References

[1] Kay AW, Xpert MTB/RIF and Xpert MTB/RIF ultra assays for active tuberculosis and rifampicin resistance in children. Cochrane Database Syst Rev. 2020;8:CD013359. 32853411 10.1002/14651858.CD013359.pub2 PMC8078611

[2] World Health Organization. Roadmap towards ending TB in children and adolescents. Geneva: WHO, 2018.

[3] Azzopardi P, Graham S. What are the most useful clinical indicators of tuberculosis in childhood? Int Child Health Rev Collab. 2008:1-8.

[4] World Health Organization. Target product profiles for tuberculosis diagnosis and detection of drug resistance. Geneva: WHO, 2024.

[5] World Health Organization. High-priority target product profiles for new tuberculosis diagnostics: report of a consensus meeting. Geneva: WHO, 2014, pp. 1-96.

[6] World Health Organization. WHO consolidated guidelines on tuberculosis: module 3: diagnosis – rapid diagnostics for tuberculosis detection: web annex 4: evidence synthesis and analysis. Geneva: WHO, 2020.

[7] Broger T, Novel lipoarabinomannan point-of-care tuberculosis test for people with HIV: a diagnostic accuracy study. Lancet Infect Dis. 2019;19(8):852-861. 31155318 10.1016/S1473-3099(19)30001-5 PMC6656794

[8] Ricks S, The potential impact of urine-LAM diagnostics on tuberculosis incidence and mortality: a modelling analysis. PLoS Med. 2020;17(12):1-20. 10.1371/journal.pmed.1003466 PMC7732057 33306694

[9] Nicol MP, Accuracy of a novel urine test, fujifilm SILVAMP tuberculosis lipoarabinomannan, for the diagnosis of pulmonary tuberculosis in children. Clin Infect Dis. 2021;72(9):e280-e288. 32761178 10.1093/cid/ciaa1052 PMC8096212

[10] Comella-Del-Barrio P, Diagnostic performance of the fujifilm silvamp tb-lam in children with presumptive tuberculosis. J Clin Med. 2021;10(9):4-11. 10.3390/jcm10091914 PMC8124322 33925008

[11] Nkereuwem E, Comparing accuracy of lipoarabinomannan urine tests for diagnosis of pulmonary tuberculosis in children from four African countries: a cross-sectional study. Lancet Infect Dis. 2021;21(3):376-384. 33316214 10.1016/S1473-3099(20)30598-3

[12] Olbrich L, FujiLAM for the diagnosis of childhood tuberculosis: a systematic review. BMJ Paediatr Open. 2022;6(1):e001447. 10.1136/bmjpo-2022-001447 PMC9280905 36053609

[13] Olbrich L, Rapid and accurate diagnosis of pediatric tuberculosis disease: a diagnostic accuracy study for pediatric tuberculosis. Pediatr Infect Dis J. 2023;42(5):353-360. 36854097 10.1097/INF.0000000000003853 PMC10097493

[14] Olbrich L, Diagnostic accuracy of a three-gene Mycobacterium tuberculosis host response cartridge using fingerstick blood for childhood tuberculosis: a multicentre prospective study in low-income and middle-income countries. Lancet Infect Dis. 2023;3099(23):1-11. 10.1016/S1473-3099(23)00491-7 PMC10808504 37918414

[15] Graham SM, Clinical case definitions for classification of intrathoracic tuberculosis in children: an update. Clin Infect Dis. 2015;61(Suppl 3):S179-S187. 10.1093/cid/civ581 PMC4583568 26409281

[16] World Medical Association. Declaration of Helsinki: ethical principles for medical research involving human subjects. Seoul, South Korea: WMA, 2008.

[17] Chabala C, Development of tuberculosis treatment decision algorithms in children below 5 years hospitalised with severe acute malnutrition in Zambia and Uganda: a prospective diagnostic cohort study. EClinicalMedicine. 2024;73:1-14. 10.1016/j.eclinm.2024.102688 PMC11245985 39007063

[18] Kroidl I, Reasons for false-positive lipoarabinomannan ELISA results in a Tanzanian population. Scand J Infect Dis. 2014;46(2):144-148. 24274710 10.3109/00365548.2013.853133

[19] Huerga H, Novel FujiLAM assay to detect tuberculosis in HIV-positive ambulatory patients in four African countries: a diagnostic accuracy study. Lancet Glob Health. 2023;11(1):e126-e135. 36521944 10.1016/S2214-109X(22)00463-6 PMC9747168

[20] Székely R, Prospective multicentre accuracy evaluation of the FUJIFILM SILVAMP TB LAM test for the diagnosis of tuberculosis in people living with HIV demonstrates lot-to-lot variability. PLoS One. 2024;19(5):e0303846. 38820372 10.1371/journal.pone.0303846 PMC11142480

[21] Adzemovic T, Diagnostic accuracy of the updated FujiLAM II assay to detect tuberculosis in outpatients with advanced HIV disease. Clin Infect Dis. 2025;81(4):e137-e142. 40130995 10.1093/cid/ciaf079 PMC12596399

[22] Connelly JT, Lipoarabinomannan point-of-care tests: evaluation with fresh samples needed. Lancet Infect Dis. 2019;19(10):1053. 10.1016/S1473-3099(19)30475-X 31559954

[23] Broger T, Tuberculosis test results using fresh versus biobanked urine samples with FujiLAM. Lancet Infect Dis. 2020;20(1):22-23. 31876492 10.1016/S1473-3099(19)30684-X

