# Supplementary material for: The use of urinary lateral-flow lipoarabinomannan assays for TB diagnosis in children
Source: IJTLD Open. 2026 May 11;3(5):327–34. doi: 10.5588/ijtldopen.25.0667 (PMC13160273; doi:10.5588/ijtldopen.25.0667)
Supplement: Supplementary file 1 [file ijtldopen25-0667_supplementarydata1.pdf]

Supplementary Information

Title: The use of urinary lateral-flow lipoarabinomannan assays for tuberculosis diagnosis in Children

List of Tables

Table S1 TB case definitions where reference standards were derived 2

Table S2: Extensive list Baseline and clinical characteristics of study participants by Diagnostic classification 3

Table S3: Baseline and clinical characteristics of study participants by Country 5

Table S4: Sensitivity, Specificity, and Predictive values of Fujifilm SILVAMP TB-LAM in children with "microbiological confirmed TB" and "Unlikely TB" (MRS) 7

Table S5: Sensitivity, Specificity, and Predictive values of Fujifilm SILVAMP TB-LAM in children with "microbiological confirmed TB" and "Unlikely TB" (MRS) 10

Table S6: Association of baseline factors and the True Positive FujiLAM results 15

Table S7: Association of baseline factors and the True Negative FujiLAM results 17

Table S8: Association of baseline factors and the True Positive AlereLAM results 19

Table S9: Association of baseline factors and the True Negative AlereLAM results 21

Table S10: STARD checklist 23

List of Figures

Figure S1: The lowess curve by country........ 14

Table S1: TB case definitions where reference standards were derived

Diagnostic classification	Description of definition
Confirmed tuberculosis	Bacteriological confirmation obtained<br>*Requires Mycobacterium tuberculosis to be confirmed (culture or Xpert® MTB/RIF (Ultra®) assay) from at least 1 specimen*
Unconfirmed tuberculosis	Bacteriological confirmation NOT obtained AND at least 2 of the following:<br>• Symptoms suggestive of TB<br>• CXR consistent with TB<br>• Recent exposure or immunologic evidence of MTB infection (TST and/or IGRA positive)<br>• Positive response to TB treatment<br>*Requires documented positive clinical response on tuberculosis treatment - no time duration specified*<br>With M. tuberculosis infection<br>*Immunological evidence of M. tuberculosis infection (TST and/or IGRA positive)*<br>Without M. tuberculosis infection<br>*No immunological evidence of M. tuberculosis infection*
Unlikely tuberculosis	Bacteriological confirmation NOT obtained AND criteria “unconfirmed TB” not met<br>With M. tuberculosis infection<br>*Immunological evidence of M. tuberculosis infection (TST and/or IGRA positive)*<br>• Without M. tuberculosis infection<br>*No immunological evidence of M. tuberculosis infection*

Table S2: Extensive list Baseline and clinical characteristics of study participants by Diagnostic classification

**Characteristics**		**Confirmed TB**	**Unconfirmed TB**	**Unlikely TB**	**Total**
		N=203	N=232	N=383	N=818
**Gender**					
Male	n (%)	113 (55.7%)	132 (56.9%)	193 (50.4%)	438 (53.5%)
**Age category**					
Age in years	Median (IQR)	7.2 (2.4-11.9)	5.6 (2.0-9.2)	5.3 (2.6-8.0)	5.5 (2.4-9.4)
<5	n (%)	74 (36.5%)	108 (46.6%)	184 (48.0%)	366 (44.7%)
5-<10 years	n (%)	53 (26.1%)	74 (31.9%)	138 (36.0%)	265 (32.4%)
10-<15 years	n (%)	76 (37.4%)	50 (21.6%)	61 (15.9%)	187 (22.9%)
**HIV status**					
HIV negative	n (%)	181 (89.2%)	166 (71.9%)	316 (82.5%)	663 (81.2%)
HIV positive	n (%)	21 (10.3%)	63 (27.3%)	54 (14.1%)	138 (16.9%)
Unknown	n (%)	1 (0.5%)	2 (0.9%)	13 (3.4%)	16 (2.0%)
**Baseline CD4 count**					
<50	n (%)	2 (12.5%)	3 (5.9%)	5 (11.6%)	10 (9.1%)
50 to <200	n (%)	3 (18.8%)	6 (11.8%)	6 (14.0%)	15 (13.6%)
200 to <500	n (%)	7 (43.8%)	16 (31.4%)	16 (37.2%)	39 (35.5%)
>= 500	n (%)	4 (25.0%)	26 (51.0%)	16 (37.2%)	46 (41.8%)
**ART naive at baseline**					
Receiving ART >1 month	n (%)	10 (47.6%)	31 (53.4%)	33 (62.3%)	74 (56.1%)
ART naive or on ART <1 month	n (%)	11 (52.4%)	27 (46.6%)	20 (37.7%)	58 (43.9%)
**Malnutrition**					
Severe acute malnutrition	n (%)	27 (13.3%)	25 (10.8%)	31 (8.1%)	83 (10.1%)
Moderate acute malnutrition	n (%)	26 (12.8%)	27 (11.6%)	43 (11.2%)	96 (11.7%)
No malnutrition	n (%)	150 (73.9%)	180 (77.6%)	309 (80.7%)	639 (78.1%)
**TST result**					
No	n (%)	73 (39.2%)	97 (45.8%)	222 (64.2%)	392 (52.7%)
Yes	n (%)	113 (60.8%)	115 (54.2%)	124 (35.8%)	352 (47.3%)
**TB contact in the last 24 months**	n (%)	85 (42.7%)	119 (53.1%)	214 (57.2%)	418 (52.4%)
**Final X-ray finding**					
Normal	n (%)	65 (34.8%)	75 (39.1%)	206 (62.0%)	346 (48.7%)
Abnormal - Likely TB	n (%)	88 (47.1%)	73 (38.0%)	52 (15.7%)	213 (30.0%)
Abnormal - Equivocal	n (%)	34 (18.2%)	44 (22.9%)	74 (22.3%)	152 (21.4%)
**Symmetrical Oedema**	n (%)				
No	n (%)	199 (98.0%)	228 (98.7%)	375 (98.2%)	802 (98.3%)
Yes	n (%)	4 (2.0%)	3 (1.3%)	7 (1.8%)	14 (1.7%)
**Mode of Urine collection**					
Catch	n (%)	111 (90.2%)	93 (92.1%)	259 (91.5%)	463 (91.3%)
Urine bag	n (%)	12 (9.8%)	8 (7.9%)	22 (7.8%)	42 (8.3%)
Other	n (%)	0 (0.0%)	0 (0.0%)	2 (0.7%)	2 (0.4%)
**LotNum**					
19001	n (%)	72 (68.6%)	77 (70.6%)	97 (49.2%)	246 (59.9%)
19003	n (%)	0 (0.0%)	2 (1.8%)	1 (0.5%)	3 (0.7%)
20001	n (%)	21 (20.0%)	2 (1.8%)	16 (8.1%)	39 (9.5%)

20003	n (%)	0 (0.0%)	2 (1.8%)	2 (1.0%)	4 (1.0%)
20004	n (%)	12 (11.4%)	26 (23.9%)	81 (41.1%)	119 (29.0%)
**Country**					
South Africa	n (%)	59 (29.1%)	58 (25.0%)	46 (12.0%)	163 (19.9%)
Tanzania	n (%)	37 (18.2%)	47 (20.3%)	112 (29.2%)	196 (24.0%)
Mozambique	n (%)	19 (9.4%)	68 (29.3%)	91 (23.8%)	178 (21.8%)
Malawi	n (%)	32 (15.8%)	55 (23.7%)	105 (27.4%)	192 (23.5%)
India	n (%)	56 (27.6%)	4 (1.7%)	29 (7.6%)	89 (10.9%)
**TB disease manifestation**					
PTB	n (%)	118 (59.9%)	168 (81.6%)	22 (56.4%)	308 (69.7%)
EPTB	n (%)	32 (16.2%)	9 (4.4%)	11 (28.2%)	52 (11.8%)
PTB & EPTB	n (%)	47 (23.9%)	29 (14.1%)	6 (15.4%)	82 (18.6%)
**FujiLAM performed**					
Bio banked	n (%)	79 (38.9%)	132 (57.6%)	195 (51.6%)	406 (50.1%)
Same Day	n (%)	124 (61.1%)	97 (42.4%)	183 (48.4%)	404 (49.9%)
**Cavitary disease**					
No	n (%)	165 (81.3%)	212 (91.4%)	370 (96.6%)	747 (91.3%)
Yes	n (%)	38 (18.7%)	20 (8.6%)	13 (3.4%)	71 (8.7%)
**Ever diagnosed with TB**	n (%)	8 (3.9%)	12 (5.2%)	9 (2.4%)	29 (3.6%)
**Ever been treated for TB**	n (%)	6 (3.0%)	11 (4.7%)	11 (2.9%)	28 (3.4%)
**Study outcome**					
Participants completed study per protocol	n (%)	183 (90.6%)	207 (89.2%)	350 (91.4%)	740 (90.6%)
Participant was lost to follow up or withdrew	n (%)	12 (5.9%)	18 (7.8%)	26 (6.8%)	56 (6.9%)
Participants died during follow up	n (%)	7 (3.5%)	7 (3.0%)	7 (1.8%)	21 (2.6%)

Table S3: Baseline and clinical characteristics of study participants by Country

	South Africa	Tanzania	Mozambique	Malawi	India	All
	**N=163**	**N=196**	**N=178**	**N=192**	**N=89**	**N=818**
**Gender**						
Male	87 (53.4%)	102 (52.0%)	88 (49.4%)	110 (57.3%)	51 (57.3%)	438 (53.5%)
**Age Median(IQR)**	2.7 (0.9-6.6)	5.2 (2.6-7.5)	5.6 (3.2-10.3)	6.7 (3.1-10.4)	10.0 (5.3-13.1)	5.5 (2.4-9.4)
<5	109 (66.9%)	95 (48.5%)	72 (40.4%)	68 (35.4%)	22 (24.7%)	366 (44.7%)
5-<10 years	33 (20.2%)	76 (38.8%)	60 (33.7%)	73 (38.0%)	23 (25.8%)	265 (32.4%)
10-<15 years	21 (12.9%)	25 (12.8%)	46 (25.8%)	51 (26.6%)	44 (49.4%)	187 (22.9%)
**HIV status**						
HIV negative	137 (84.0%)	185 (94.9%)	133 (74.7%)	122 (63.5%)	86 (96.6%)	663 (81.2%)
HIV positive	25 (15.3%)	10 (5.1%)	38 (21.3%)	63 (32.8%)	2 (2.2%)	138 (16.9%)
Unknown	1 (0.6%)	0 (0.0%)	7 (3.9%)	7 (3.6%)	1 (1.1%)	16 (2.0%)
**Baseline CD4 count**						
<50	2 (9.1%)	0 (0.0%)	1 (3.6%)	7 (14.0%)	0 (0.0%)	10 (9.1%)
50 to <200	5 (22.7%)	1 (12.5%)	2 (7.1%)	7 (14.0%)	0 (0.0%)	15 (13.6%)
200 to <500	10 (45.5%)	3 (37.5%)	13 (46.4%)	11 (22.0%)	2 (100.0%)	39 (35.5%)
>= 500	5 (22.7%)	4 (50.0%)	12 (42.9%)	25 (50.0%)	0 (0.0%)	46 (41.8%)
**ART naive at baseline**						
Receiving ART >1 month	6 (24.0%)	7 (87.5%)	18 (52.9%)	43 (68.3%)	0 (0.0%)	74 (56.1%)
ART naive or on ART <1 month	19 (76.0%)	1 (12.5%)	16 (47.1%)	20 (31.7%)	2 (100.0%)	58 (43.9%)
**TST result**						
TST Positive	66 (54.1%)	130 (66.3%)	69 (46.3%)	55 (28.8%)	32 (37.2%)	352 (47.3%)
**TB contact in the last 24 months**						
Yes	57 (35.2%)	165 (88.7%)	117 (69.2%)	73 (38.2%)	6 (6.7%)	418 (52.4%)
**Final Xray finding**						
Normal	36 (22.2%)	114 (64.0%)	63 (56.2%)	95 (55.6%)	38 (43.2%)	346 (48.7%)
Abnormal - Likely TB	90 (55.6%)	33 (18.5%)	24 (21.4%)	36 (21.1%)	30 (34.1%)	213 (30.0%)
Abnormal - Equivocal	36 (22.2%)	31 (17.4%)	25 (22.3%)	40 (23.4%)	20 (22.7%)	152 (21.4%)
**Symmetrical oedema**						
No	161 (99.4%)	191 (97.4%)	177 (100.0%)	188 (97.9%)	85 (95.5%)	802 (98.3%)
Yes	1 (0.6%)	5 (2.6%)	0 (0.0%)	4 (2.1%)	4 (4.5%)	14 (1.7%)
**Mode of Urine collection**						
Catch	45 (52.9%)	123 (99.2%)	111 (99.1%)	100 (100.0%)	84 (97.7%)	463 (91.3%)
Urine bag	38 (44.7%)	1 (0.8%)	1 (0.9%)	0 (0.0%)	2 (2.3%)	42 (8.3%)
Other	2 (2.4%)	0 (0.0%)	0 (0.0%)	0 (0.0%)	0 (0.0%)	2 (0.4%)
**LotNum**						
19001	0 (.%)	0 (.%)	72 (55.4%)	124 (64.6%)	50 (56.2%)	246 (59.9%)
19003	0 (.%)	0 (.%)	3 (2.3%)	0 (0.0%)	0 (0.0%)	3 (0.7%)
20001	0 (.%)	0 (.%)	0 (0.0%)	0 (0.0%)	39 (43.8%)	39 (9.5%)
20003	0 (.%)	0 (.%)	4 (3.1%)	0 (0.0%)	0 (0.0%)	4 (1.0%)
20004	0 (.%)	0 (.%)	51 (39.2%)	68 (35.4%)	0 (0.0%)	119 (29.0%)
**Clinical case definition**						
Confirmed TB	59 (36.2%)	37 (18.9%)	19 (10.7%)	32 (16.7%)	56 (62.9%)	203 (24.8%)

Unconfirmed TB	58 (35.6%)	47 (24.0%)	68 (38.2%)	55 (28.6%)	4 (4.5%)	232 (28.4%)
Unlikely TB	46 (28.2%)	112 (57.1%)	91 (51.1%)	105 (54.7%)	29 (32.6%)	383 (46.8%)
**TB disease manifestation**						
PTB	72 (60.5%)	73 (91.2%)	75 (92.6%)	77 (81.1%)	11 (16.4%)	308 (69.7%)
EPTB	11 (9.2%)	1 (1.2%)	2 (2.5%)	1 (1.1%)	37 (55.2%)	52 (11.8%)
PTB & EPTB	36 (30.3%)	6 (7.5%)	4 (4.9%)	17 (17.9%)	19 (28.4%)	82 (18.6%)
**FujiLAM performed**						
Bio banked	42 (25.8%)	164 (85.0%)	111 (64.2%)	81 (42.2%)	8 (9.0%)	406 (50.1%)
Same Day	121 (74.2%)	29 (15.0%)	62 (35.8%)	111 (57.8%)	81 (91.0%)	404 (49.9%)
**Cavitary disease**						
No	138 (84.7%)	185 (94.4%)	167 (93.8%)	180 (93.8%)	77 (86.5%)	747 (91.3%)
Yes	25 (15.3%)	11 (5.6%)	11 (6.2%)	12 (6.2%)	12 (13.5%)	71 (8.7%)
**Ever diagnosed with TB**	7 (4.3%)	3 (1.6%)	10 (5.6%)	7 (3.7%)	2 (2.2%)	29 (3.6%)
**Ever been treated for TB**	6 (3.7%)	3 (1.5%)	9 (5.1%)	9 (4.7%)	1 (1.1%)	28 (3.4%)
**Study outcome**						
Visits completed per protocol	145 (89.0%)	190 (97.4%)	158 (88.8%)	163 (84.9%)	84 (94.4%)	740 (90.6%)
Discontinued	17 (10.4%)	3 (1.5%)	20 (11.2%)	14 (7.3%)	2 (2.2%)	56 (6.9%)
Died before the end of study	1 (0.6%)	2 (1.0%)	0 (0.0%)	15 (7.8%)	3 (3.4%)	21 (2.6%)

Table S4: Sensitivity, Specificity, and Predictive values of Fujifilm SILVAMP TB-LAM in children with "microbiological confirmed TB" and "Unlikely TB" (MRS)

SRS	Test	N	TP	FN	FP	TN	Sensitivity (95% CI)	p-value	Specificity (95% CI)	PPV (95% CI)	NPV (95% CI)
**Overall**	FujiLAM	586	63	140	39	344	31.0% (24.7-37.9)	P<0.0001	89.8% (86.3-92.7)	61.8% (51.6-71.2)	71.1% (66.8-75.1)
	AlereLAM	586	27	176	28	355	13.3% (9.0-18.8)		92.7% (89.6-95.1)	49.1% (35.4-62.9)	66.9% (62.7-70.8)
**Age**											
<5	FujiLAM	258	24	50	23	161	32.4% (22.0-44.3)	0.04	87.5% (81.8-91.9)	51.1% (36.1-65.9)	76.3% (70.0-81.9)
	AlereLAM	258	15	59	22	162	20.3% (11.8-31.2)		88.0% (82.5-92.4)	40.5% (24.8-57.9)	73.3% (67.0-79.0)
5-<10 years	FujiLAM	191	9	44	10	128	17.0% (8.1-29.8)	0.45	92.8% (87.1-96.5)	47.4% (24.4-71.1)	74.4% (67.2-80.8)
	AlereLAM	191	6	47	3	135	11.3% (4.3-23.0)		97.8% (93.8-99.5)	66.7% (29.9-92.5)	74.2% (67.2-80.4)
10-<15 years	FujiLAM	137	30	46	6	55	39.5% (28.4-51.4)	P<0.0001	90.2% (79.8-96.3)	83.3% (67.2-93.6)	54.5% (44.2-64.4)
	AlereLAM	137	6	70	3	58	7.9% (3.0-16.4)		95.1% (86.3-99.0)	66.7% (29.9-92.5)	45.3% (36.5-54.3)
**Gender**											
Female	FujiLAM	280	34	56	18	172	37.8% (27.8-48.6)	P<0.0001	90.5% (85.4-94.3)	65.4% (50.9-78.0)	75.4% (69.3-80.9)
	AlereLAM	280	14	76	16	174	15.6% (8.8-24.7)		91.6% (86.7-95.1)	46.7% (28.3-65.7)	69.6% (63.5-75.2)
Male	FujiLAM	306	29	84	21	172	25.7% (17.9-34.7)	0.002	89.1% (83.8-93.1)	58.0% (43.2-71.8)	67.2% (61.1-72.9)
	AlereLAM	306	13	100	12	181	11.5% (6.3-18.9)		93.8% (89.4-96.7)	52.0% (31.3-72.2)	64.4% (58.5-70.0)
**HIV Status**											
HIV Positive	FujiLAM	75	5	16	11	43	23.8% (8.2-47.2)	0.13	79.6% (66.5-89.4)	31.2% (11.0-58.7)	72.9% (59.7-83.6)
	AlereLAM	75	1	20	3	51	4.8% (0.1-23.8)		94.4% (84.6-98.8)	25.0% (0.6-80.6)	71.8% (59.9-81.9)
HIV Negative	FujiLAM	415	49	107	23	236	31.4% (24.2-39.3)	P<0.0001	91.1% (87.0-94.3)	68.1% (56.0-78.6)	68.8% (63.6-73.7)
	AlereLAM	415	20	136	21	238	12.8% (8.0-19.1)		91.9% (87.9-94.9)	48.8% (32.9-64.9)	63.6% (58.5-68.5)
**Baseline CD4 count**											
HIV pos CD4 <+200	FujiLAM	16	1	4	1	10	20.0% (0.5-71.6)	-	90.9% (58.7-99.8)	50.0% (1.3-98.7)	71.4% (41.9-91.6)
	AlereLAM	16	0	5	1	10	0.0% (0.0-52.2)		90.9% (58.7-99.8)	0.0% (0.0-97.5)	66.7% (38.4-88.2)
HIV pos CD4 >200	FujiLAM	43	3	8	8	24	27.3% (6.0-61.0)	-	75.0% (56.6-88.5)	27.3% (6.0-61.0)	75.0% (56.6-88.5)
	AlereLAM	43	1	10	1	31	9.1% (0.2-41.3)		96.9% (83.8-99.9)	50.0% (1.3-98.7)	75.6% (59.7-87.6)
**ART at baseline**											

ART Naïve	FujiLAM	31	5	6	7	13	45.5% (16.7-76.6)	0.125	65.0% (40.8-84.6)	41.7% (15.2-72.3)	68.4% (43.4-87.4)
	AlereLAM	31	1	10	1	19	9.1% (0.2-41.3)		95.0% (75.1-99.9)	50.0% (1.3-98.7)	65.5% (45.7-82.1)
ART Ongoing	FujiLAM	45	0	11	5	29	0.0% (0.0-28.5)	1	85.3% (68.9-95.0)	0.0% (0.0-52.2)	72.5% (56.1-85.4)
	AlereLAM	45	1	10	2	32	9.1% (0.2-41.3)		94.1% (80.3-99.3)	33.3% (0.8-90.6)	76.2% (60.5-87.9)
**Malnutrition**											
Severe acute malnutrition	FujiLAM	58	10	17	3	28	37.0% (19.4-57.6)	0.02	90.3% (74.2-98.0)	76.9% (46.2-95.0)	62.2% (46.5-76.2)
	AlereLAM	58	3	24	4	27	11.1% (2.4-29.2)		87.1% (70.2-96.4)	42.9% (9.9-81.6)	52.9% (38.5-67.1)
No malnutrition	FujiLAM	459	48	102	27	282	32.0% (24.6-40.1)	P<0.001	91.3% (87.5-94.2)	64.0% (52.1-74.8)	73.4% (68.7-77.8)
	AlereLAM	459	21	129	22	287	14.0% (8.9-20.6)		92.9% (89.4-95.5)	48.8% (33.3-64.5)	69.0% (64.3-73.4)
Moderate acute malnutrition	FujiLAM	69	5	21	9	34	19.2% (6.6-39.4)	0.63	79.1% (64.0-90.0)	35.7% (12.8-64.9)	61.8% (47.7-74.6)
	AlereLAM	69	3	23	2	41	11.5% (2.4-30.2)		95.3% (84.2-99.4)	60.0% (14.7-94.7)	64.1% (51.1-75.7)
**TB disease manifestation**											
Non Severe TB	FujiLAM	54	4	38	2	10	9.5% (2.7-22.6)	1	83.3% (51.6-97.9)	66.7% (22.3-95.7)	20.8% (10.5-35.0)
	AlereLAM	54	5	37	2	10	11.9% (4.0-25.6)		83.3% (51.6-97.9)	71.4% (29.0-96.3)	21.3% (10.7-35.7)
PTB	FujiLAM	140	43	75	6	16	36.4% (27.8-45.8)	P<0.001	72.7% (49.8-89.3)	87.8% (75.2-95.4)	17.6% (10.4-27.0)
	AlereLAM	140	18	100	4	18	15.3% (9.3-23.0)		81.8% (59.7-94.8)	81.8% (59.7-94.8)	15.3% (9.3-23.0)
EPTB	FujiLAM	43	4	28	1	10	12.5% (3.5-29.0)	0.38	90.9% (58.7-99.8)	80.0% (28.4-99.5)	26.3% (13.4-43.1)
	AlereLAM	43	1	31	1	10	3.1% (0.1-16.2)		90.9% (58.7-99.8)	50.0% (1.3-98.7)	24.4% (12.4-40.3)
PTB_EPTB	FujiLAM	53	16	31	2	4	34.0% (20.9-49.3)	0.02	66.7% (22.3-95.7)	88.9% (65.3-98.6)	11.4% (3.2-26.7)
	AlereLAM	53	8	39	0	6	17.0% (7.6-30.8)		100% (54.1-100)	100% (63.1-100)	13.3% (5.1-26.8)
**TST result**											
TST Positive	FujiLAM	237	33	80	10	114	29.2% (21.0-38.5)	P<0.001	91.9% (85.7-96.1)	76.7% (61.4-88.2)	58.8% (51.5-65.8)
	AlereLAM	237	11	102	9	115	9.7% (5.0-16.8)		92.7% (86.7-96.6)	55.0% (31.5-76.9)	53.0% (46.1-59.8)
TST Negative	FujiLAM	295	19	54	23	199	26.0% (16.5-37.6)	0.08	89.6% (84.9-93.3)	45.2% (29.8-61.3)	78.7% (73.1-83.5)
	AlereLAM	295	11	62	17	205	15.1% (7.8-25.4)		92.3% (88.0-95.5)	39.3% (21.5-59.4)	76.8% (71.2-81.7)
**Final X-ray finding**											
Normal	FujiLAM	271	8	57	20	186	12.3% (5.5-22.8)	1	90.3% (85.4-94.0)	28.6% (13.2-48.7)	76.5% (70.7-81.7)
	AlereLAM	271	8	57	15	191	12.3% (5.5-22.8)		92.7% (88.3-95.9)	34.8% (16.4-57.3)	77.0% (71.3-82.1)
Abnormal - Likely TB	FujiLAM	140	38	50	7	45	43.2% (32.7-54.2)	P<0.001	86.5% (74.2-94.4)	84.4% (70.5-93.5)	47.4% (37.0-57.9)
	AlereLAM	140	13	75	4	48	14.8% (8.1-23.9)		92.3% (81.5-97.9)	76.5% (50.1-93.2)	39.0% (30.4-48.2)

Abnormal - Equivocal	FujiLAM	108	11	23	8	66	32.4% (17.4-50.5)	0.04	89.2% (79.8-95.2)	57.9% (33.5-79.7)	74.2% (63.8-82.9)
	AlereLAM	108	4	30	6	68	11.8% (3.3-27.5)		91.9% (83.2-97.0)	40.0% (12.2-73.8)	69.4% (59.3-78.3)
**Country**											
South Africa	FujiLAM	105	30	29	12	34	50.8% (37.5-64.1)	0.0001	73.9% (58.9-85.7)	71.4% (55.4-84.3)	54.0% (40.9-66.6)
	AlereLAM	105	14	45	4	42	23.7% (13.6-36.6)		91.3% (79.2-97.6)	77.8% (52.4-93.6)	48.3% (37.4-59.2)
Tanzania	FujiLAM	149	6	31	8	104	16.2% (6.2-32.0)	0.125	92.9% (86.4-96.9)	42.9% (17.7-71.1)	77.0% (69.0-83.8)
	AlereLAM	149	1	36	3	109	2.7% (0.1-14.2)		97.3% (92.4-99.4)	25.0% (0.6-80.6)	75.2% (67.3-82.0)
Mozambique	FujiLAM	110	6	13	7	84	31.6% (12.6-56.6)	0.22	92.3% (84.8-96.9)	46.2% (19.2-74.9)	86.6% (78.2-92.7)
	AlereLAM	110	2	17	14	77	10.5% (1.3-33.1)		84.6% (75.5-91.3)	12.5% (1.6-38.3)	81.9% (72.6-89.1)
Malawi	FujiLAM	137	9	23	10	95	28.1% (13.7-46.7)	0.29	90.5% (83.2-95.3)	47.4% (24.4-71.1)	80.5% (72.2-87.2)
	AlereLAM	137	5	27	5	100	15.6% (5.3-32.8)		95.2% (89.2-98.4)	50.0% (18.7-81.3)	78.7% (70.6-85.5)
India	FujiLAM	85	12	44	2	27	21.4% (11.6-34.4)	0.04	93.1% (77.2-99.2)	85.7% (57.2-98.2)	38.0% (26.8-50.3)
	AlereLAM	85	5	51	2	27	8.9% (3.0-19.6)		93.1% (77.2-99.2)	71.4% (29.0-96.3)	34.6% (24.2-46.2)
**LAM performed**											
Same Day	FujiLAM	307	45	79	26	157	36.3% (27.8-45.4)	P<0.001	85.8% (79.9-90.5)	63.4% (51.1-74.5)	66.5% (60.1-72.5)
	AlereLAM	307	18	106	14	169	14.5% (8.8-22.0)		92.3% (87.5-95.8)	56.2% (37.7-73.6)	61.5% (55.4-67.2)
Bio banked	FujiLAM	274	18	61	13	182	22.8% (14.1-33.6)	0.06	93.3% (88.9-96.4)	58.1% (39.1-75.5)	74.9% (69.0-80.2)
	AlereLAM	274	9	70	14	181	11.4% (5.3-20.5)		92.8% (88.2-96.0)	39.1% (19.7-61.5)	72.1% (66.1-77.6)
**Mode of Urine Collection**											
Catch	FujiLAM	370	29	82	19	240	26.1% (18.2-35.3)	0.003	92.7% (88.8-95.5)	60.4% (45.3-74.2)	74.5% (69.4-79.2)
	AlereLAM	370	11	100	15	244	9.9% (5.1-17.0)		94.2% (90.6-96.7)	42.3% (23.4-63.1)	70.9% (65.8-75.7)
Urine bag	FujiLAM	34	7	5	3	19	58.3% (27.7-84.8)	0.06	86.4% (65.1-97.1)	70.0% (34.8-93.3)	79.2% (57.8-92.9)
	AlereLAM	34	2	10	3	19	16.7% (2.1-48.4)		86.4% (65.1-97.1)	40.0% (5.3-85.3)	65.5% (45.7-82.1)
Other	FujiLAM	180	27	53	16	84	33.8% (23.6-45.2)	-	84.0% (75.3-90.6)	62.8% (46.7-77.0)	61.3% (52.6-69.5)
	AlereLAM	180	14	66	10	90	17.5% (9.9-27.6)		90.0% (82.4-95.1)	58.3% (36.6-77.9)	57.7% (49.5-65.6)
**LotNum**											
19001	FujiLAM	169	22	50	14	83	30.6% (20.2-42.5)	-	85.6% (77.0-91.9)	61.1% (43.5-76.9)	62.4% (53.6-70.7)
	AlereLAM	169	9	63	10	87	12.5% (5.9-22.4)		89.7% (81.9-94.9)	47.4% (24.4-71.1)	58.0% (49.7-66.0)
19003	FujiLAM	1	0	0	0	1		-	100% (2.5-100)		100% (2.5-100)
	AlereLAM	1	0	0	1	0			0.0% (0.0-97.5)	0.0% (0.0-97.5)	
20001	FujiLAM	37	2	19	0	16	9.5% (1.2-30.4)	-	100% (79.4-100)	100% (15.8-100)	45.7% (28.8-63.4)
	AlereLAM	37	2	19	1	15	9.5% (1.2-30.4)		93.8% (69.8-99.8)	66.7% (9.4-99.2)	44.1% (27.2-62.1)
20003	FujiLAM	2	0	0	0	2		-	100% (15.8-100)		100% (15.8-100)
	AlereLAM	2	0	0	0	2			100% (15.8-100)		100% (15.8-100)

20004	FujiLAM	93	2	10	3	78	16.7% (2.1-48.4)	-	96.3% (89.6-99.2)	40.0% (5.3-85.3)	88.6% (80.1-94.4)
	AlereLAM	93	1	11	7	74	8.3% (0.2-38.5)		91.4% (83.0-96.5)	12.5% (0.3-52.7)	87.1% (78.0-93.4)

Table S5: Sensitivity, Specificity, and Predictive values of Fujifilm SILVAMP TB-LAM in children using a Composite Reference Standard

CRS	Test	N	TP	FN	FP	TN	Sensitivity (95% CI)	Specificity (95% CI)	PPV (95% CI)	NPV (95% CI)
**Overall**	FujiLAM	818	92	343	39	344	21.1% (17.4-25.3)	89.8% (86.3-92.7)	70.2% (61.6-77.9)	50.1% (46.3-53.9)
	AlereLAM	818	50	385	28	355	11.5% (8.7-14.9)	92.7% (89.6-95.1)	64.1% (52.4-74.7)	48.0% (44.3-51.6)
**Age**										
<5	FujiLAM	366	40	142	23	161	22.0% (16.2-28.7)	87.5% (81.8-91.9)	63.5% (50.4-75.3)	53.1% (47.3-58.9)
	AlereLAM	366	32	150	22	162	17.6% (12.3-23.9)	88.0% (82.5-92.4)	59.3% (45.0-72.4)	51.9% (46.2-57.6)
5-<10 years	FujiLAM	265	17	110	10	128	13.4% (8.0-20.6)	92.8% (87.1-96.5)	63.0% (42.4-80.6)	53.8% (47.2-60.2)
	AlereLAM	265	7	120	3	135	5.5% (2.2-11.0)	97.8% (93.8-99.5)	70.0% (34.8-93.3)	52.9% (46.6-59.2)
10-<15 years	FujiLAM	187	35	91	6	55	27.8% (20.2-36.5)	90.2% (79.8-96.3)	85.4% (70.8-94.4)	37.7% (29.8-46.1)
	AlereLAM	187	11	115	3	58	8.7% (4.4-15.1)	95.1% (86.3-99.0)	78.6% (49.2-95.3)	33.5% (26.5-41.1)
**Gender**										
Female	FujiLAM	380	48	142	18	172	25.3% (19.3-32.1)	90.5% (85.4-94.3)	72.7% (60.4-83.0)	54.8% (49.1-60.4)
	AlereLAM	380	26	164	16	174	13.7% (9.1-19.4)	91.6% (86.7-95.1)	61.9% (45.6-76.4)	51.5% (46.0-56.9)
Male	FujiLAM	438	44	201	21	172	18.0% (13.4-23.3)	89.1% (83.8-93.1)	67.7% (54.9-78.8)	46.1% (41.0-51.3)
	AlereLAM	438	24	221	12	181	9.8% (6.4-14.2)	93.8% (89.4-96.7)	66.7% (49.0-81.4)	45.0% (40.1-50.0)
**HIV Status**										
HIV Positive	FujiLAM	138	10	74	11	43	11.9% (5.9-20.8)	79.6% (66.5-89.4)	47.6% (25.7-70.2)	36.8% (28.0-46.2)
	AlereLAM	138	3	81	3	51	3.6% (0.7-10.1)	94.4% (84.6-98.8)	50.0% (11.8-88.2)	38.6% (30.3-47.5)
HIV Negative	FujiLAM	558	67	232	23	236	22.4% (17.8-27.6)	91.1% (87.0-94.3)	74.4% (64.2-83.1)	50.4% (45.8-55.0)
	AlereLAM	558	38	261	21	238	12.7% (9.2-17.0)	91.9% (87.9-94.9)	64.4% (50.9-76.4)	47.7% (43.2-52.2)
**Baseline CD4 count**										
HIV pos CD4 <+200	FujiLAM	25	4	10	1	10	28.6% (8.4-58.1)	90.9% (58.7-99.8)	80.0% (28.4-99.5)	50.0% (27.2-72.8)
	AlereLAM	25	2	12	1	10	14.3% (1.8-42.8)	90.9% (58.7-99.8)	66.7% (9.4-99.2)	45.5% (24.4-67.8)

HIV pos CD4 >200	FujiLAM	85	4	49	8	24	7.5% (2.1-18.2)	75.0% (56.6-88.5)	33.3% (9.9-65.1)	32.9% (22.3-44.9)
	AlereLAM	85	1	52	1	31	1.9% (0.0-10.1)	96.9% (83.8-99.9)	50.0% (1.3-98.7)	37.3% (27.0-48.7)
**ART at baseline**										
ART Naïve	FujiLAM	58	7	31	7	13	18.4% (7.7-34.3)	65.0% (40.8-84.6)	50.0% (23.0-77.0)	29.5% (16.8-45.2)
	AlereLAM	58	2	36	1	19	5.3% (0.6-17.7)	95.0% (75.1-99.9)	66.7% (9.4-99.2)	34.5% (22.2-48.6)
ART Ongoing	FujiLAM	76	3	39	5	29	7.1% (1.5-19.5)	85.3% (68.9-95.0)	37.5% (8.5-75.5)	42.6% (30.7-55.2)
	AlereLAM	76	2	40	2	32	4.8% (0.6-16.2)	94.1% (80.3-99.3)	50.0% (6.8-93.2)	44.4% (32.7-56.6)
**Malnutrition**										
Severe acute malnutrition	FujiLAM	83	15	37	3	28	28.8% (17.1-43.1)	90.3% (74.2-98.0)	83.3% (58.6-96.4)	43.1% (30.8-56.0)
	AlereLAM	83	7	45	4	27	13.5% (5.6-25.8)	87.1% (70.2-96.4)	63.6% (30.8-89.1)	37.5% (26.4-49.7)
No malnutrition	FujiLAM	639	70	260	27	282	21.2% (16.9-26.0)	91.3% (87.5-94.2)	72.2% (62.1-80.8)	52.0% (47.7-56.3)
	AlereLAM	639	37	293	22	287	11.2% (8.0-15.1)	92.9% (89.4-95.5)	62.7% (49.1-75.0)	49.5% (45.3-53.6)
Moderate acute malnutrition	FujiLAM	96	7	46	9	34	13.2% (5.5-25.3)	79.1% (64.0-90.0)	43.8% (19.8-70.1)	42.5% (31.5-54.1)
	AlereLAM	96	6	47	2	41	11.3% (4.3-23.0)	95.3% (84.2-99.4)	75.0% (34.9-96.8)	46.6% (35.9-57.5)
**TB disease manifestation**										
Non Severe TB	FujiLAM	139	14	113	2	10	11.0% (6.2-17.8)	83.3% (51.6-97.9)	87.5% (61.7-98.4)	8.1% (4.0-14.4)
	AlereLAM	139	17	110	2	10	13.4% (8.0-20.6)	83.3% (51.6-97.9)	89.5% (66.9-98.7)	8.3% (4.1-14.8)
PTB	FujiLAM	308	66	220	6	16	23.1% (18.3-28.4)	72.7% (49.8-89.3)	91.7% (82.7-96.9)	6.8% (3.9-10.8)
	AlereLAM	308	34	252	4	18	11.9% (8.4-16.2)	81.8% (59.7-94.8)	89.5% (75.2-97.1)	6.7% (4.0-10.3)
EPTB	FujiLAM	52	5	36	1	10	12.2% (4.1-26.2)	90.9% (58.7-99.8)	83.3% (35.9-99.6)	21.7% (10.9-36.4)
	AlereLAM	52	2	39	1	10	4.9% (0.6-16.5)	90.9% (58.7-99.8)	66.7% (9.4-99.2)	20.4% (10.2-34.3)
PTB_EPTB	FujiLAM	82	20	56	2	4	26.3% (16.9-37.7)	66.7% (22.3-95.7)	90.9% (70.8-98.9)	6.7% (1.8-16.2)
	AlereLAM	82	12	64	0	6	15.8% (8.4-26.0)	100% (54.1-100)	100% (73.5-100)	8.6% (3.2-17.7)
**TST result**										
TST Positive	FujiLAM	352	46	182	10	114	20.2% (15.2-26.0)	91.9% (85.7-96.1)	82.1% (69.6-91.1)	38.5% (32.9-44.3)
	AlereLAM	352	23	205	9	115	10.1% (6.5-14.8)	92.7% (86.7-96.6)	71.9% (53.3-86.3)	35.9% (30.7-41.5)
TST Negative	FujiLAM	392	32	138	23	199	18.8% (13.2-25.5)	89.6% (84.9-93.3)	58.2% (44.1-71.3)	59.1% (53.6-64.3)
	AlereLAM	392	19	151	17	205	11.2% (6.9-16.9)	92.3% (88.0-95.5)	52.8% (35.5-69.6)	57.6% (52.3-62.8)
**Final X-ray finding**										
Normal	FujiLAM	346	14	126	20	186	10.0% (5.6-16.2)	90.3% (85.4-94.0)	41.2% (24.6-59.3)	59.6% (53.9-65.1)

	AlereLAM	346	19	121	15	191	13.6% (8.4-20.4)	92.7% (88.3-95.9)	55.9% (37.9-72.8)	61.2% (55.6-66.7)
Abnormal - Likely TB	FujiLAM	213	49	112	7	45	30.4% (23.4-38.2)	86.5% (74.2-94.4)	87.5% (75.9-94.8)	28.7% (21.7-36.4)
	AlereLAM	213	16	145	4	48	9.9% (5.8-15.6)	92.3% (81.5-97.9)	80.0% (56.3-94.3)	24.9% (18.9-31.6)
Abnormal - Equivocal	FujiLAM	152	15	63	8	66	19.2% (11.2-29.7)	89.2% (79.8-95.2)	65.2% (42.7-83.6)	51.2% (42.2-60.1)
	AlereLAM	152	7	71	6	68	9.0% (3.7-17.6)	91.9% (83.2-97.0)	53.8% (25.1-80.8)	48.9% (40.4-57.5)
**Country**										
South Africa	FujiLAM	163	39	78	12	34	33.3% (24.9-42.6)	73.9% (58.9-85.7)	76.5% (62.5-87.2)	30.4% (22.0-39.8)
	AlereLAM	163	18	99	4	42	15.4% (9.4-23.2)	91.3% (79.2-97.6)	81.8% (59.7-94.8)	29.8% (22.4-38.1)
Tanzania	FujiLAM	196	10	74	8	104	11.9% (5.9-20.8)	92.9% (86.4-96.9)	55.6% (30.8-78.5)	58.4% (50.8-65.8)
	AlereLAM	196	7	77	3	109	8.3% (3.4-16.4)	97.3% (92.4-99.4)	70.0% (34.8-93.3)	58.6% (51.2-65.8)
Mozambique	FujiLAM	178	16	71	7	84	18.4% (10.9-28.1)	92.3% (84.8-96.9)	69.6% (47.1-86.8)	54.2% (46.0-62.2)
	AlereLAM	178	11	76	14	77	12.6% (6.5-21.5)	84.6% (75.5-91.3)	44.0% (24.4-65.1)	50.3% (42.1-58.5)
Malawi	FujiLAM	192	15	72	10	95	17.2% (10.0-26.8)	90.5% (83.2-95.3)	60.0% (38.7-78.9)	56.9% (49.0-64.5)
	AlereLAM	192	9	78	5	100	10.3% (4.8-18.7)	95.2% (89.2-98.4)	64.3% (35.1-87.2)	56.2% (48.6-63.6)
India	FujiLAM	89	12	48	2	27	20.0% (10.8-32.3)	93.1% (77.2-99.2)	85.7% (57.2-98.2)	36.0% (25.2-47.9)
	AlereLAM	89	5	55	2	27	8.3% (2.8-18.4)	93.1% (77.2-99.2)	71.4% (29.0-96.3)	32.9% (22.9-44.2)
**LAM performed**										
Same Day	FujiLAM	404	63	158	26	157	28.5% (22.7-34.9)	85.8% (79.9-90.5)	70.8% (60.2-79.9)	49.8% (44.2-55.5)
	AlereLAM	404	26	195	14	169	11.8% (7.8-16.8)	92.3% (87.5-95.8)	65.0% (48.3-79.4)	46.4% (41.2-51.7)
Bio banked	FujiLAM	406	29	182	13	182	13.7% (9.4-19.1)	93.3% (88.9-96.4)	69.0% (52.9-82.4)	50.0% (44.7-55.3)
	AlereLAM	406	24	187	14	181	11.4% (7.4-16.5)	92.8% (88.2-96.0)	63.2% (46.0-78.2)	49.2% (44.0-54.4)
**Mode of Urine collection**										
Catch	FujiLAM	463	36	168	19	240	17.6% (12.7-23.6)	92.7% (88.8-95.5)	65.5% (51.4-77.8)	58.8% (53.9-63.6)
	AlereLAM	463	23	181	15	244	11.3% (7.3-16.4)	94.2% (90.6-96.7)	60.5% (43.4-76.0)	57.4% (52.6-62.2)
Urine bag	FujiLAM	42	8	12	3	19	40.0% (19.1-63.9)	86.4% (65.1-97.1)	72.7% (39.0-94.0)	61.3% (42.2-78.2)
	AlereLAM	42	3	17	3	19	15.0% (3.2-37.9)	86.4% (65.1-97.1)	50.0% (11.8-88.2)	52.8% (35.5-69.6)
Other	FujiLAM	311	48	163	16	84	22.7% (17.3-29.0)	84.0% (75.3-90.6)	75.0% (62.6-85.0)	34.0% (28.1-40.3)
	AlereLAM	311	24	187	10	90	11.4% (7.4-16.5)	90.0% (82.4-95.1)	70.6% (52.5-84.9)	32.5% (27.0-38.4)
**LotNum**										
19001	FujiLAM	246	34	115	14	83	22.8% (16.3-30.4)	85.6% (77.0-91.9)	70.8% (55.9-83.0)	41.9% (35.0-49.1)
	AlereLAM	246	13	136	10	87	8.7% (4.7-14.5)	89.7% (81.9-94.9)	56.5% (34.5-76.8)	39.0% (32.6-45.8)

19003	FujiLAM	3	1	1	0	1	50.0% (1.3-98.7)	100% (2.5-100)	100% (2.5-100)	50.0% (1.3-98.7)
	AlereLAM	3	1	1	1	0	50.0% (1.3-98.7)	0.0% (0.0-97.5)	50.0% (1.3-98.7)	0.0% (0.0-97.5)
20001	FujiLAM	39	2	21	0	16	8.7% (1.1-28.0)	100% (79.4-100)	100% (15.8-100)	43.2% (27.1-60.5)
	AlereLAM	39	2	21	1	15	8.7% (1.1-28.0)	93.8% (69.8-99.8)	66.7% (9.4-99.2)	41.7% (25.5-59.2)
20003	FujiLAM	4	0	2	0	2	0.0% (0.0-84.2)	100% (15.8-100)		50.0% (6.8-93.2)
	AlereLAM	4	0	2	0	2	0.0% (0.0-84.2)	100% (15.8-100)		50.0% (6.8-93.2)
20004	FujiLAM	119	5	33	3	78	13.2% (4.4-28.1)	96.3% (89.6-99.2)	62.5% (24.5-91.5)	70.3% (60.9-78.6)
	AlereLAM	119	7	31	7	74	18.4% (7.7-34.3)	91.4% (83.0-96.5)	50.0% (23.0-77.0)	70.5% (60.8-79.0)

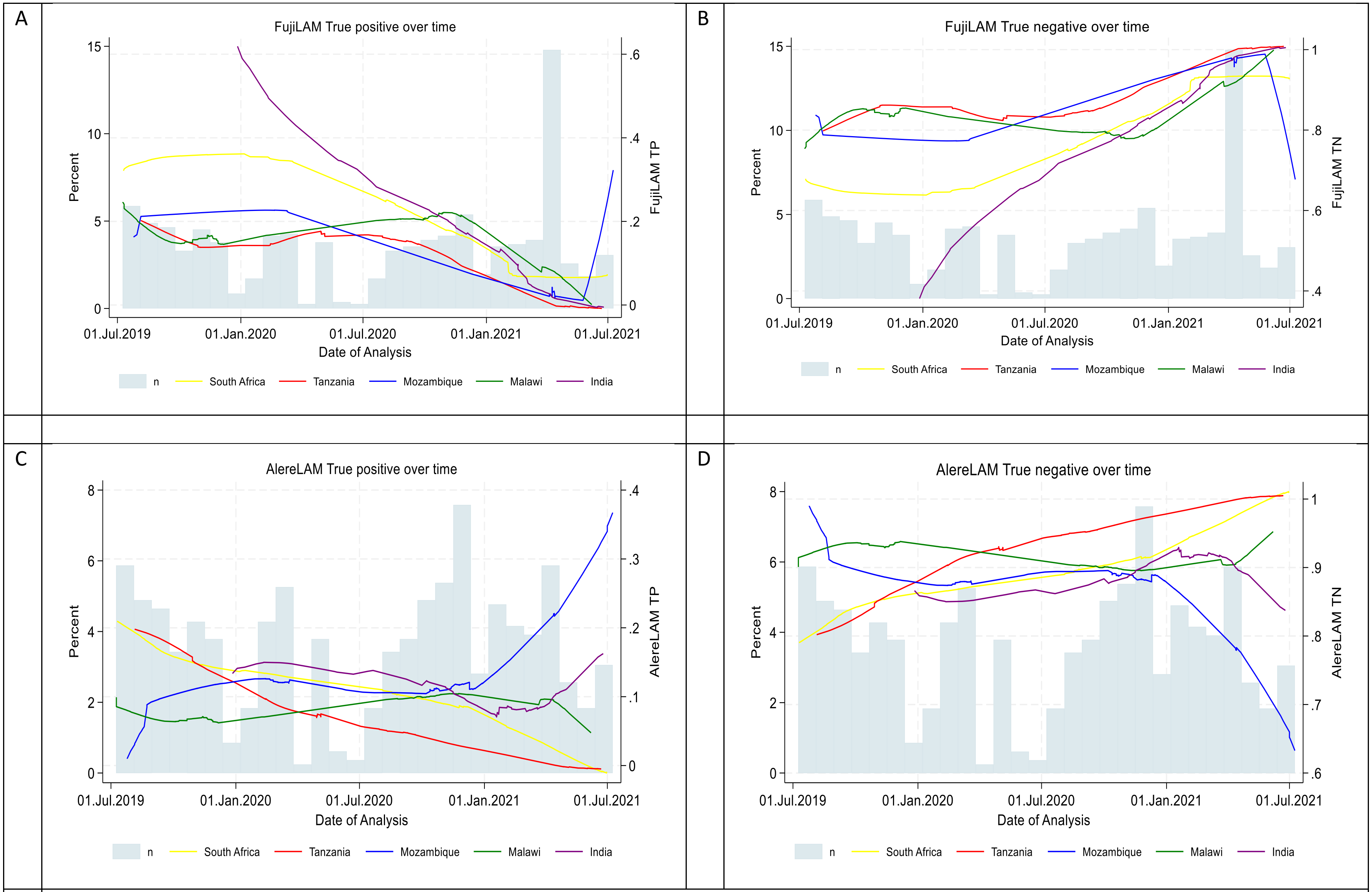

Figure S1: The lowess curve by country. A & B shows lowess curve for the sensitivity and specificity of FujiLAM over time, respectively. The graph shows that sensitivity decreases over time in every country, while specificity increases over time for South Africa and India, remaining constant for the other countries. C & D show the lowess curve for the sensitivity and specificity of AlereLAM over time, respectively. The graph shows that sensitivity slowly decreases over time in every country, while specificity remains constant across the countries.

Table S6: Association of baseline factors and the True Positive FujiLAM results

	True Positive FujiLAM					
	No	Yes	COR (95% CI)	p-value	AOR (95% CI)	p-value
	N=140	N=63				
**Gender of child**						
Female	56 (40.0%)	34 (54.0%)	ref		ref	
Male	84 (60.0%)	29 (46.0%)	0.59 (0.32-1.10)	0.09	0.56 (0.17-1.80)	0.33
**Age category**						
0-5 yr	50 (35.7%)	24 (38.1%)	ref		ref	
5-10 yr	44 (31.4%)	9 (14.3%)	0.57 (0.23-1.45)	0.24	0.31 (0.06-1.61)	0.16
10-15 yr	46 (32.9%)	30 (47.6%)	2.69 (1.16-6.26)	0.02	1.26 (0.34-4.69)	0.73
**HIV status**						
HIV negative	123 (88.5%)	58 (92.1%)	ref			
HIV positive	16 (11.5%)	5 (7.9%)	0.53 (0.17-1.60)	0.26		
**Baseline CD4 count**						
CD4 count <200/ul	4 (33.3%)	1 (25.0%)	ref			
CD4 count >200/ul	8 (66.7%)	3 (75.0%)	1.5 (0.12-19.44)	0.76		
**Final Result TST/Mantoux**						
Negative	54 (40.3%)	19 (36.5%)	ref		ref	
Positive	80 (59.7%)	33 (63.5%)	1.13 (0.56-2.27)	0.73	1.34 (0.46-3.90)	0.60
**TB contact in the last 24 months**						
No	79 (57.2%)	35 (57.4%)	ref			
Yes	59 (42.8%)	26 (42.6%)	1.06 (0.53-2.15)	0.87		
**Cavitary disease**						
No	124 (88.6%)	41 (65.1%)	ref			
Yes	16 (11.4%)	22 (34.9%)	3.98 (1.86-8.50)	P<0.0001		
**Malnutrition**						
SAM	17 (12.1%)	10 (15.9%)	ref		ref	
MAM	21 (15.0%)	5 (7.9%)	0.32 (0.09-1.19)	0.09	0.22 (0.03-1.53)	0.13
No MAM	102 (72.9%)	48 (76.2%)	0.58 (0.23-1.45)	0.24	0.37 (0.10-1.33)	0.13
**Oedema**						
No	137 (97.9%)	62 (98.4%)	ref			
Yes	3 (2.1%)	1 (1.6%)	0.80 (0.07-8.46)	0.79		
**Final Xray finding**						
Normal	57 (43.8%)	8 (14.0%)	ref			
Abnormal - Likely TB	50 (38.5%)	38 (66.7%)	4.81 (2.01-11.52)	0.001		
Abnormal - Equivocal	23 (17.7%)	11 (19.3%)	3.40 (1.18-9.76)	0.02		
**Time to positivity (MGIT)**						
TTP_0to9	6 (9.4)	16 (33.3)	ref			
TTP_10to14	12 (18.8)	12 (25.0)	0.26 (0.06-1.09)	0.07		
TTP_15to26	17 (26.6)	8 (16.7)	0.11 (0.02-0.48)	0.003		
TTP_27to42	29 (45.3)	12 (25.6)	0.09 (0.02-0.39)	0.001		
**FujiLAM Performed**						
Bio banked	61 (43.6)	18 (28.6)	ref			
Same Day	79 (56.4)	45 (71.4)	1.99 (0.96-4.13)	0.04		

**TB Manifestation**						
PTB	75 (56.0)	43 (68.3)	ref		ref	
EPTB	28 (20.9)	4 (6.3)	0.22 (0.06-0.76)	0.02	0.65(0.16-2.67)	0.55
PTB & EPTB	31 (23.1)	16 (25.4)	0.71 (0.32-1.58)	0.40	0.60 (0.16-2.25)	0.45
**Mode of Urine collection**						
Catch	82 (94.3)	29 (80.6)	ref			
Urine bag	5 (5.7)	7 (19.4)	3.96 (1.16-13.45)	0.03		
**LotNum**						
19001	50 (63.3%)	22 (84.6%)	ref		ref	
20001	19 (24.1%)	2 (7.7%)	0.24 (0.05-1.12)	0.07	0.09 (0.01-0.84)	0.04
20004	10 (12.7%)	2 (7.7%)	0.45 (0.09-2.25)	0.33	0.23 (0.04-1.45)	0.12
**No of TB Symptoms**						
0	16 (11.4%)	2 (3.2%)	ref			
1	44 (31.4%)	15 (23.8%)	3.18 (0.63-16.08)	0.16		
2	59 (42.1%)	24 (38.1%)	3.81 (0.78-18.72)	0.10		
3	21 (15.0%)	22 (34.9%)	7.99 (1.57-40.69)	0.01		

Table S7: Association of baseline factors and the True Negative FujiLAM results

	True Negative FujiLAM					
	No	Yes	COR (95% CI)	p- value	AOR (95% CI)	p-value
	N=39	N=344				
**Gender of child**						
Female	18 (46.2%)	172 (50.0%)	ref			
Male	21 (53.8%)	172 (50.0%)	0.87 (0.44-1.71)	0.68	0.82 (0.24-2.77)	0.75
**Age category**						
0-5 yr	23 (59.0%)	161 (46.8%)	ref			
5-10 yr	10 (25.6%)	128 (37.2%)	1.65 (0.74-3.68)	0.22	3.47 (0.87-13.81)	0.07
10-15 yr	6 (15.4%)	55 (16.0%)	1.18 (0.44-3.15)	0.75	7.93 (1.22-51.61)	0.03
**HIV status**						
HIV negative	28 (71.8%)	288 (87.0%)	ref			
HIV positive	11 (28.2%)	43 (13.0%)	0.41(0.18-0.92)	0.03	0.12 (0.03-0.53)	0.01
**Baseline CD4 count**						
CD4 count <200/ul	1 (11.1%)	10 (29.4%)	ref			
CD4 count >200/ul	8 (88.9%)	24 (70.6%)	0.3 (0.03-2.72)	0.29		
**Final Result TST/Mantoux**						
Negative	23 (69.7%)	199 (63.6%)	ref		ref	
Positive	10(30.3%)	114 (36.4%)	1.20 (0.51-2.78)	0.68	0.38(0.10-1.54)	0.18
**TB contact in the last 24 months**						
No	21 (55.3%)	139 (41.4%)	ref			
Yes	17 (44.7%)	197 (58.6%)	1.50 (0.64-3.48)	0.35		
**Cavitary disease**						
No	37 (94.9%)	333 (96.8%)	ref			
Yes	2 (5.1%)	11 (3.2%)	0.70 (0.14-3.39)	0.65		
**Malnutrition**						
SAM	3 (7.7%)	28 (8.1%)	ref			
MAM	9 (23.1%)	34 (9.9%)	0.46 (0.11-1.93)	0.29		
No MAM	27 (69.2%)	282 (82.0%)	1.22 (0.34-4.36)	0.77		
**Final X-ray finding**						
Normal	20 (57.1%)	186 (62.6%)	ref			
Abnormal - Likely TB	7 (20.0%)	45 (15.2%)	0.90 (0.33-2.49)	0.85		

Abnormal - Equivocal	8 (22.9%)	66 (22.2%)	1.04 (0.42-2.59)	0.93		
**FujiLAM Performed**						
Bio banked	13 (33.3)	182(53.7)	ref			
Same Day	26 (66.7)	157 (46.3)	0.46 (0.21-0.98)	0.05		
**Mode of Urine collection**						
Catch	19 (82.6)	240 (92.3)	ref			
Urine bag	3 (13.0)	19 (7.3)	0.50(0.14-1.85)	0.30		
**LotNum**						
19001	14 (82.4%)	83 (46.1%)	ref			
20001	0 (0.0%)	16 (8.9%)				
20003	0 (0.0%)	2 (1.1%)				
20004	3 (17.6%)	78 (43.3%)	4.39 (1.21-15.85)	0.02	4.27 (1.08-16.83)	0.04
**No TB symptoms**						
0	1 (2.6%)	11 (3.2%)	ref			
1	15 (38.5%)	143 (41.6%)	0.69 (0.08-6.02)	0.74		
2	16 (41.0%)	164 (47.7%)	0.79 (0.09-7.80)	0.83		
3	7 (17.9%)	26 (7.6%)	0.31 (0.03-2.90)	0.30		

Table S8: Association of baseline factors and the True Positive AlereLAM results

	True Positive AlereLAM					
	No	Yes	COR (95% CI)	p-value	AOR (95% CI)	p-value
	N=176	N=27				
**Gender of child**						
Female	76 (43.2%)	14 (51.9%)	ref		ref	
Male	100 (56.8%)	13 (48.1%)	0.74 (0.32-1.70)	0.48	0.51 (0.20-1.30)	0.16
**Age category**						
0-5 yr	59 (33.5%)	15 (55.6%)	ref		ref	
5-10 yr	47 (26.7%)	6 (22.2%)	0.55 (0.19-1.57)	0.26	0.39 (0.13-1.22)	0.10
10-15 yr	70 (39.8%)	6 (22.2%)	0.38 (0.13-1.14)	0.08	0.24 (0.07-0.77)	0.02
**HIV status**						
HIV negative	155 (88.6%)	26 (96.3%)	ref			
HIV positive	20 (11.4%)	1 (3.7%)	0.22 (0.03-1.82)	0.16		
**Final Result TST/Mantoux**						
Negative	62 (37.6%)	12 (50.0%)	ref			
Positive	103 (62.4%)	12 (50.0%)	0.58 (0.23-1.47)	0.25		
**TB contact in the last 24 months**						
No	96 (55.8%)	18 (66.7%)	ref			
Yes	76 (44.2%)	9 (33.3%)	0.63 (0.25-1.58)	0.32		
**Cavitary disease**						
No	84 (73.0%)	13 (65.0%)	ref			
Yes	31 (27.0%)	7 (35.0%)	1.49 (0.57-3.92)			
**Malnutrition**						
SAM	24 (14.5%)	3 (11.1%)	ref			
MAM	23 (13.9%)	3 (11.1%)	0.94 (0.17-5.30)	0.94		
No MAM	118 (71.5%)	21 (77.8%)	1.08 (0.28-4.14)	0.91		
**Final X-ray finding**						
Normal	57 (35.2%)	8 (32.0%)	ref		ref	
Abnormal - Likely TB	75 (46.3%)	13 (52.0%)	1.04 (0.39-2.80)	0.94	0.95 (0.29-3.07)	0.93
Abnormal - Equivocal	30 (18.5%)	4 (16.0%)	0.88 (0.24-3.25)	0.85	0.75 (0.19-3.01)	0.69
**Time to positivity (MGIT)**						
TTP_0to9	17 (17.9)	5 (29.4)	Ref			

TTP_10to14	20 (21.1)	4 (23.5)	0.57 (0.12-2.79)	0.49		
TTP_15to26	21 (22.1)	4 (23.5)	0.55 (0.12-2.60)	0.45		
TTP_27to42	37 (38.9)	4 923.5)	0.31 (0.07-1.45)	0.14		
**FujiLAM Performed**						
Bio banked	70 (39.8)	9 (33.3)	ref			
Same Day	106 (60.2)	18 (66.7)	1.15 (0.44-2.98)	0.77		
**TB Manifestation**						
PTB	100 (58.8)	18 (66.7)	ref			
EPTB	31 (18.2)	1 (3.7)	0.17 (0.02-1.43)	0.10		
PTB & EPTB	39 (22.9)	8 (29.6)	0.97 (0.36-2.59)	0.95		
**Mode of Urine collection**						
Catch	100 (90.9)	11 (84.6)	ref			
Urine bag	10 (9.1)	2 (15.4)	1.82 (0.35-9.38)	0.48		
**LotNum**						
19001	63 (67.7%)	9 (75.0%)	ref			
20001	19 (20.4%)	2 (16.7%)	0.74 (0.15-3.71)	0.71		
20004	11 (11.8%)	1 (8.3%)	0.64 (0.07-5.53)	0.68		
**No of TB Symptoms**						
0	18 (10.2%)	0 (0.0%)				
1	53 (30.1%)	6 (22.2%)	ref			
2	71 (40.3%)	12 (44.4%)	1.52 (0.53-4.39)	0.44	1.28 (0.41-4.02)	0.68
3	34 (19.3%)	9 (33.3%)	1.88 (0.58-6.13)	0.29	1.61 (0.44-5.91)	0.47

Table S9: Association of baseline factors and the True Negative AlereLAM results

	True Negative AlereLAM					
	No	Yes	COR (95% CI)	p-value	AOR (95% CI)	p-value
	N=28	N=355				
**Gender of child**						
Female	16 (57.1%)	174 (49.0%)	ref		ref	
Male	12 (42.9%)	181 (51.0%)	1.31 (0.59-2.89)	0.51	1.56 (0.65-3.72)	0.32
**Age category**						
0-5 yr	22 (78.6%)	162 (45.6%)	ref		ref	
5-10 yr	3 (10.7%)	135 (38.0%)	6.00 (1.74-20.72)	0.004	8.72 (1.97-38.56)	0.004
10-15 yr	3 (10.7%)	58 (16.3%)	2.75 (0.78-9.78)	0.12	2.53 (0.70-9.15)	0.16
**HIV status**						
HIV negative	23 (88.5%)	293 (85.2%)	ref			
HIV positive	3 (11.5%)	51 (14.8%)	1.42 (0.40-5.05)	0.59		
**Baseline CD4 count**						
CD4 count <200/ul	1 (50.0%)	10 (24.4%)	ref			
CD4 count >200/ul	1 (50.0%)	31 (75.6%)	3.1 (0.18-54.24)	0.44		
**Final Result TST/Mantoux**						
Negative	17 (65.4%)	205 (64.1%)	ref		ref	
Positive	9 (34.6%)	115 (35.9%)	1.04 (0.43-2.54)	0.92	1.24 (0.49-3.09)	0.65
**TB contact in the last 24 months**						
No	12 (44.4%)	148 (42.7%)	ref			
Yes	15 (55.6%)	199 (57.3%)	1.20 (0.48-3.01)	0.70		
**Malnutrition**						
SAM	4 (14.3%)	27 (7.6%)	ref			
MAM	2 (7.1%)	41 (11.5%)	3.22 (0.54-19.38)	0.20		
No MAM	22 (78.6%)	287 (80.8%)	2.07 (0.64-6.70)	0.23		
**Final X-ray finding**						
Normal	15 (60.0%)	191 (62.2%)	ref			
Abnormal - Likely TB	4 (16.0%)	48 (15.6%)	0.97 (0.29-3.18)	0.96		
Abnormal - Equivocal	6 (24.0%)	68 (22.1%)	0.93 (0.34-2.55)	0.88		
**FujiLAM Performed**						
Bio banked	14 (50.0)	181 (51.7)	ref			

Same Day	14 (50.0)	169 (48.3)	0.92 (0.40-2.12)	0.84		
**Mode of Urine collection**						
Catch	15 (83.3)	244 (92.1)	ref			
Urine bag	3 (16.7)	19 (7.2)	0.35 (0.07-1.81)	0.21		
**LotNum**						
19001	10 (52.6%)	87 (48.9%)	ref			
19003	1 (5.3%)	0				
20001	1 (5.3%)	15 (8.4%)	1.68 (0.16-17.87)	0.67		
20003	0 (0.0%)	2 (1.1%)				
20004	7 (36.8%)	74 (41.6%)	1.32 (0.46-3.76)	0.61		
**No of TB Symptoms**						
0	1 (3.6%)	11 (3.1%)	ref			
1	7 (25.0%)	151 (42.5%)	2.90 (0.29-28.61)	0.36		
2	13 (46.4%)	167 (47.0%)	1.34 (0.15-12.09)	0.79		
3	7 (25.0%)	26 (7.3%)	0.35 (0.04 -3.45)	0.37		
